# CD93 maintains endothelial barrier function and limits metastatic dissemination

**DOI:** 10.1172/jci.insight.169830

**Published:** 2024-03-05

**Authors:** Kalyani Vemuri, Beatriz de Alves Pereira, Patricia Fuenzalida, Yelin Subashi, Stefano Barbera, Luuk van Hooren, Marie Hedlund, Fredrik Pontén, Cecilia Lindskog, Anna-Karin Olsson, Roberta Lugano, Anna Dimberg

**Affiliations:** 1 Department of Immunology, Genetics and Pathology, Rudbeck Laboratory, Science for Life Laboratory, and; 2 Department of Medical Biochemistry and Microbiology, Uppsala University Biomedical Center, Science for Life Laboratory, Uppsala University, Uppsala, Sweden.

**Keywords:** Oncology, Vascular biology, Endothelial cells, Extracellular matrix, Melanoma

## Abstract

Compromised vascular integrity facilitates extravasation of cancer cells and promotes metastatic dissemination. CD93 has emerged as a target for antiangiogenic therapy, but its importance for vascular integrity in metastatic cancers has not been evaluated. Here, we demonstrate that CD93 participates in maintaining the endothelial barrier and reducing metastatic dissemination. Primary melanoma growth was hampered in *CD93^–/–^* mice, but metastatic dissemination was increased and associated with disruption of adherens and tight junctions in tumor endothelial cells and elevated expression of matrix metalloprotease 9 at the metastatic site. CD93 directly interacted with vascular endothelial growth factor receptor 2 (VEGFR2) and its absence led to VEGF-induced hyperphosphorylation of VEGFR2 in endothelial cells. Antagonistic anti-VEGFR2 antibody therapy rescued endothelial barrier function and reduced the metastatic burden in *CD93^–/–^* mice to wild-type levels. These findings reveal a key role of CD93 in maintaining vascular integrity, which has implications for pathological angiogenesis and endothelial barrier function in metastatic cancer.

## Abstract 2

CD93 maintains endothelial barrier function and reduces metastatic dissemination by limiting VEGFR2 activation.

## Graphical abstract

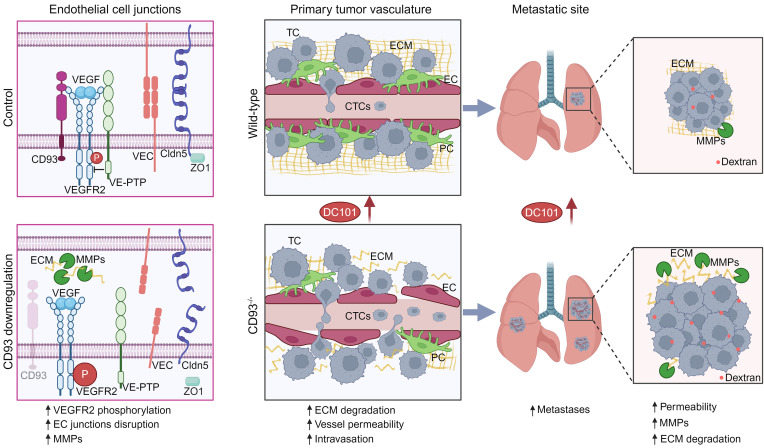

## Introduction

Tumor blood vessels are functionally abnormal due to a sustained release of proangiogenic factors in the tumor microenvironment (1). Intratumoral hypoxia induces expression of vascular endothelial growth factor (VEGF) and reduces endothelial barrier function (2, 3). VEGF signaling through its cognate receptor VEGFR2 dismantles endothelial cell junctions and promotes the degradation of the vascular basement membrane through secretion of matrix metalloproteases (MMPs), thereby facilitating cancer cell intravasation and metastasis (4–6). Activation of VEGFR2 is modulated by VEGF coreceptors such as neuropilins and heparan sulfate proteoglycans, and through interactions with other receptors such as ephrinB2, integrins, and phosphatases (7). Vascular endothelial protein tyrosine phosphatase (VE-PTP) regulates the activity of VEGFR2 and the adherens junction protein VE-cadherin in endothelial junctions in a TIE2-dependent manner (8, 9). Tumor-derived factors alter gene transcription in endothelial cells, which contributes to the aberrant structure and dysfunctionality of the vessels (10–12). The consequence of specific gene regulation in modulating VEGFR2 signaling and vascular stability is still poorly understood, and needs further investigation to identify proteins suitable for therapeutic targeting.

CD93 has emerged as a potential target for antiangiogenic therapy, owing to its association with tumor angiogenesis in human cancers (13). CD93 is a receptor for insulin-like growth factor binding protein 7 (IGFBP7, also known as angiomodulin), which is secreted by tumor vessels and contributes to vascular abnormalities (14). In addition, CD93 binds to multimerin-2 (MMRN2) in the extracellular matrix (ECM), which protects CD93 from proteolytic cleavage (15–17). CD93 interacts with and promotes the activation of integrin β1, an important regulator of fibronectin fibrillogenesis, and knockdown of CD93 in endothelial cells disrupts organization of fibronectin networks (15). The CD93-interacting proteins IGFBP7, MMRN2, and integrin β1 have all been implicated in modulating VEGFR2 signaling. Treatment with IGPBP7 inhibits VEGF-induced signaling in endothelial cells, and MMRN2 can sequester VEGF and thereby reduce VEGFR2 activation (18, 19). VEGFR2 interaction with VEGF bound to ECM promotes complex formation with integrin β1 in focal adhesions, and is instead associated with prolonged receptor activation (20). Interestingly, CD93 has recently been identified as a putative substrate of the VEGFR2 phosphatase, VE-PTP (21), but the potential crosstalk between the CD93 and VEGFR2 signaling pathways has not been assessed. CD93 regulates endothelial cytoskeletal organization and junctional stability, suggesting a crucial role of CD93 in maintaining vascular integrity (15, 22). However, the molecular mechanisms involved and its potential importance for maintaining the endothelial barrier in metastatic cancer has not been evaluated.

Here, we investigated the role of CD93 in regulating endothelial junctions and maintaining vascular integrity in metastatic melanoma. We demonstrate that CD93 forms a complex with VEGFR2 in endothelial cells and that the absence of CD93 enhances VEGF-induced VEGFR2 phosphorylation, which was associated with a disruption of VEGFR2–VE-PTP interactions. CD93 deficiency in melanoma-bearing mice was associated with reduced endothelial barrier function in the primary tumor, enhanced expression of MMP9, and increased metastatic spread, which was reversed to wild-type levels when VEGFR2 signaling was inhibited. Collectively, our data indicate that CD93 deficiency leads to a hyperresponsiveness of VEGFR2 to VEGF stimulation, reducing vascular integrity and enhancing metastatic dissemination.

## Results

### CD93, MMRN2, and fibronectin are coexpressed in the vasculature of primary metastatic tumors and metastatic lesions.

**Figure 1:**
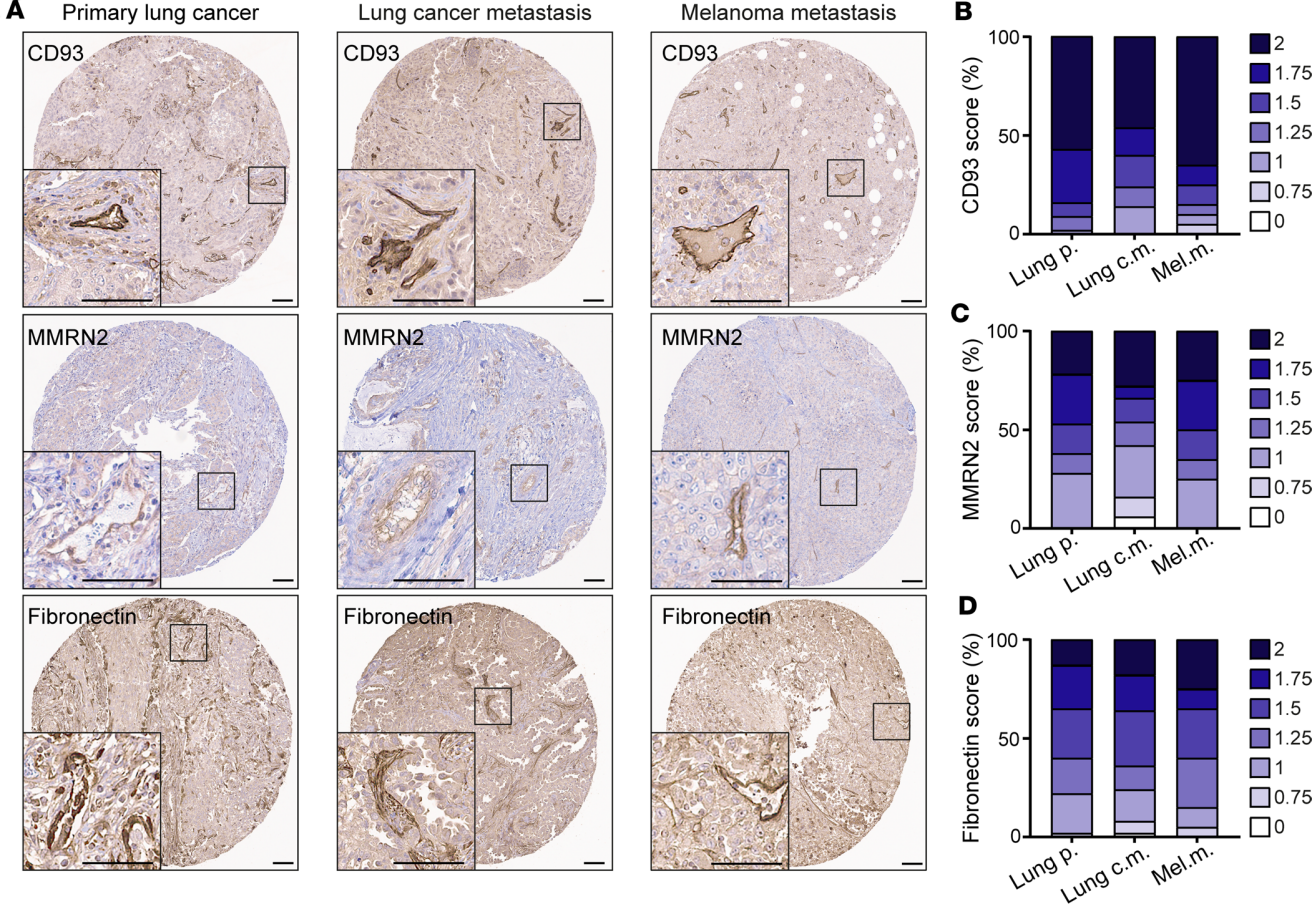
CD93, MMRN2, and fibronectin are highly expressed in the blood vessels of primary tumors and metastases. (**A**) Immunohistochemical staining of CD93, MMRN2, and fibronectin in human tissue microarrays of primary lung cancer (*n* = 60), metastases originating from lung cancer (*n* = 50), and melanoma metastases (*n* = 20). Scale bars: 100 μm. Graphs represent the average of a semiquantitative scoring of CD93^+^ (**B**), MMRN2^+^ (**C**), and fibronectin^+^ (**D**) vessels performed in tumor cores of each patient by 2 researchers in a blinded fashion on a scale of 0 to 2 (0 = no vessel staining, 1 = medium intensity, and 2 = high intensity). Lung p., lung primary tumors; Lung c.m., lung cancer metastases; Mel.m., melanoma metastases.

CD93 is generally expressed in tumor endothelial cells in many types of primary tumors, but its expression pattern in metastases has not been evaluated. We scored the fraction of blood vessels that expressed CD93 and its partners MMRN2 and fibronectin in tissue microarrays (TMAs) containing tumor tissue cores from primary and metastatic lung tumors as well as melanoma metastases (23). CD93, MMRN2, and fibronectin expression was observed in the vasculature of primary lung cancer as well as in lung metastases and melanoma metastases (Figure 1A). A semiquantitative scoring method was used to determine the staining intensity of positive vessels in each core (Supplemental Figure 1, A–C; supplemental material available online with this article; https://doi.org/10.1172/jci.insight.169830DS1). CD93, MMRN2, and fibronectin were generally highly expressed in blood vessels of both primary tumors and metastatic tissues (Figure 1, B–D).

Notably, high CD93 scores in the vasculature of lung cancer metastasis were associated with higher MMRN2 and fibronectin scores in the same tumor tissue (Supplemental Figure 1, D and E). A similar association between a high CD93 score and higher MMRN2 and fibronectin scoring was also observed in primary lung cancer (Supplemental Figure 1, F and G). Taken together, the data indicate that CD93 is expressed in the vasculature of primary tumors and metastatic lesions and its expression correlated with that of MMRN2 and fibronectin.

### CD93 is required for maintaining tumor vessel integrity in murine melanoma.

**Figure 2:**
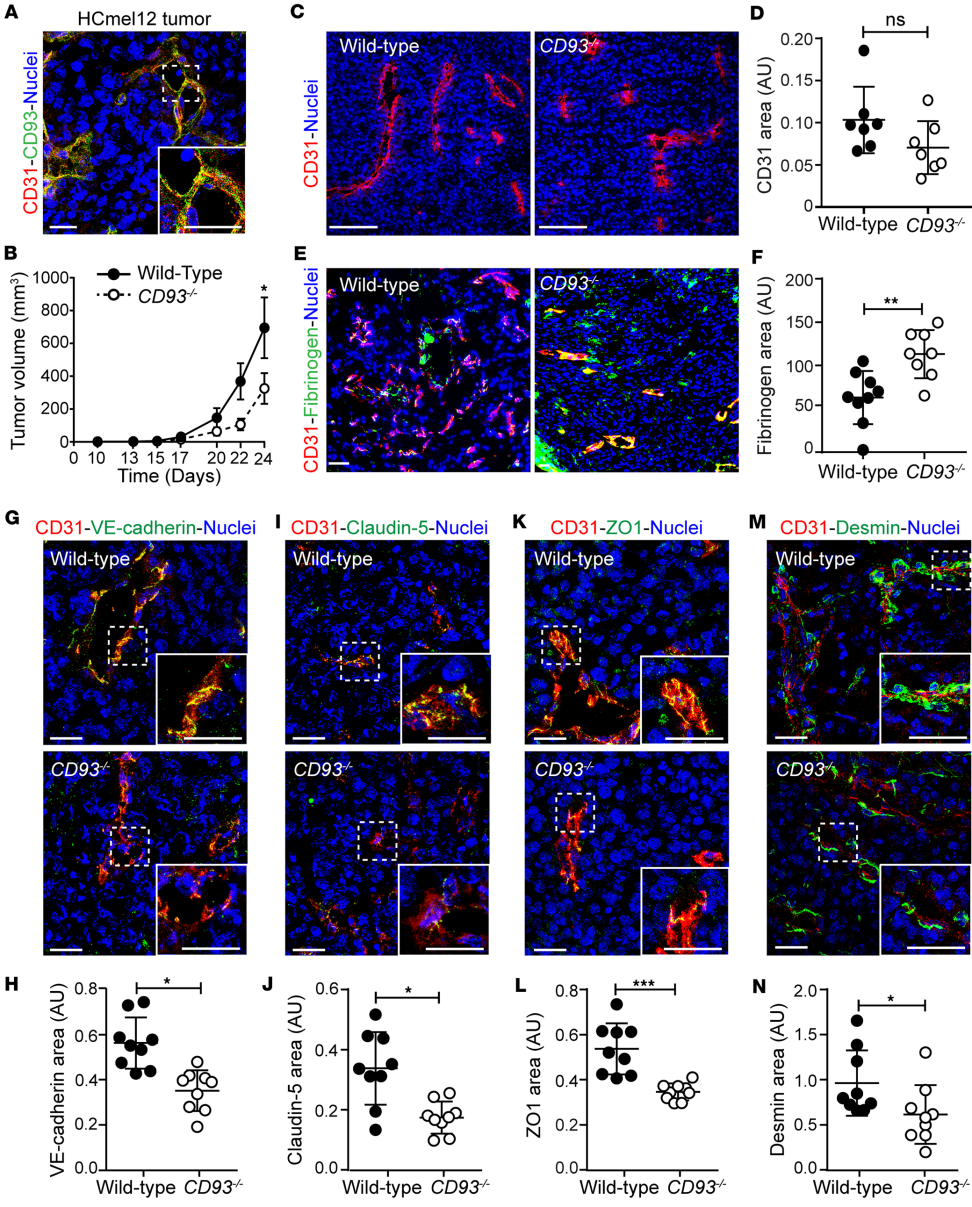
CD93 deficiency impairs subcutaneous HCmel12 melanoma growth and tumor vascular integrity. (**A**) HCmel12 tumor stained for CD93 (green), CD31 (red), and with Hoechst (blue). Scale bars: 20 μm. (**B**) Tumor growth in wild-type and *CD93^–/–^* mice (*n* = 16/group). *P < 0.05 by 2-way ANOVA. (**C**) Representative images of tumor vessels stained with Hoechst (blue) and for CD31 (red). Scale bars: 100 μm. (**D**) Quantification of CD31^+^ area in wild-type and *CD93^–/–^* tumors (*n* = 7/group, 6–8 fields of view/sample). (**E**) Representative images of fibrinogen leakage (green) and vessels (CD31, red). Scale bar: 150 μm. (**F**) Quantification of tumor vessel leakage in wild-type and *CD93^–/–^* mice (tumor tilescans, *n* = 8–9/group). Nuclei are visualized by Hoechst (blue). AU, arbitrary units; NS, not significant. **P < 0.01 by 2-tailed *t* test. Immunofluorescence images for the endothelial junction markers VE-cadherin (green) (**G**), claudin-5 (green) (**I**), ZO1 (green) (**K**), and desmin (green) (**M**) in HCmel12 tumors. (**H**, **J**, **L**, and **N**) Quantification graphs represent the area covered by the analyzed endothelial junction markers normalized to the CD31^+^ area in wild-type (*n* = 9, minimum of 5 fields of view/sample) and *CD93^–/–^* (*n* = 9 minimum of 5 fields of view/sample) tumors. Scale bars: 20 μm. *P < 0.05, ***P < 0.001 by 2-tailed *t* test. Values represent mean ± SEM.

We have previously shown that CD93 is involved in the regulation of endothelial barrier function and vessel maturation in an experimental model of glioblastoma (15, 22). To investigate whether CD93 affects vascular integrity in metastatic tumors, we employed the murine HCmel12 melanoma model (24). CD93 colocalized with the endothelial marker CD31, indicating a predominant expression in tumor endothelial cells (Figure 2A). Similar to our previous observations in glioma and fibrosarcoma models (18), HCmel12 tumor growth was significantly decreased in *CD93^–/–^* mice as compared with wild-type mice (Figure 2B).

Tumor vessel parameters, including CD31-positive area, vascular volume, length, and diameter were similar in tumors grown in wild-type and *CD93^–/–^* mice (Figure 2, C and D, and Supplemental Figure 2, A–C). Consistent with this, tumor hypoxia, as analyzed either by using hypoxyprobe or Glut1 staining (Supplemental Figure 2, D–G) in HCmel12 melanomas, did not differ between the 2 groups. However, vascular permeability, indicated by fibrinogen leakage, was significantly increased in HCmel12 tumors from *CD93^–/–^* mice, indicating a disruption of vascular integrity (Figure 2, E and F).

Endothelial cell-cell junctions are crucial for maintaining vascular barrier properties and their disruption has been associated with tumor progression and metastatic spread (4, 25). CD93 has been associated with regulation of endothelial junctions in physiological and pathological conditions (22, 26, 27). Therefore, we investigated whether CD93 deficiency affects endothelial junctions in the melanoma-associated vessels.

A significant reduction in the expression of the adherens junction protein VE-cadherin was observed by immunofluorescent staining in the HCmel12 tumor vessels of *CD93^–/–^* mice (Figure 2, G and H). Similarly, the levels of the tight junction molecules claudin-5 and zonula occludens-1 (ZO1) were also significantly decreased in *CD93^–/–^* tumor vessels as compared with the wild-type group (Figure 2, I–L). The mRNA levels of VE-cadherin, claudin-5, and ZO1 were similar in HCmel12 tumors from wild-type and *CD93^–/–^* mice, indicating that loss of CD93 regulates these junctional proteins through posttranscriptional mechanisms (Supplemental Figure 3, A–C).

Pericytes are important for preserving vascular integrity, and loss of pericytes from tumor vessels has been associated with increased metastatic dissemination (28). Interestingly, the relative area of the vasculature covered by desmin-positive pericytes was significantly reduced in *CD93^–/–^* HCmel12 tumor vessels as compared with wild-type tumor vessels (Figure 2, M and N).

To determine whether the loss of vascular integrity in response to CD93 deficiency was also evident in other melanoma models, wild-type and *CD93^–/–^* mice were subcutaneously injected with B16F10 melanoma cells. Similarly to the HCmel12 model, CD93 was highly expressed in the vasculature of B16F10 melanomas, and tumor growth was decreased in *CD93^–/–^* mice (Supplemental Figure 4, A and B). While the vascular area was not affected by CD93 deficiency, VE-cadherin was downregulated and there was a trend toward a reduction in ZO1 area (Supplemental Figure 4, C–H).

Altogether, these data show that CD93 deficiency impairs vascular integrity in melanoma.

### CD93 deficiency facilitates transendothelial migration of melanoma cells and promotes metastases.

**Figure 3:**
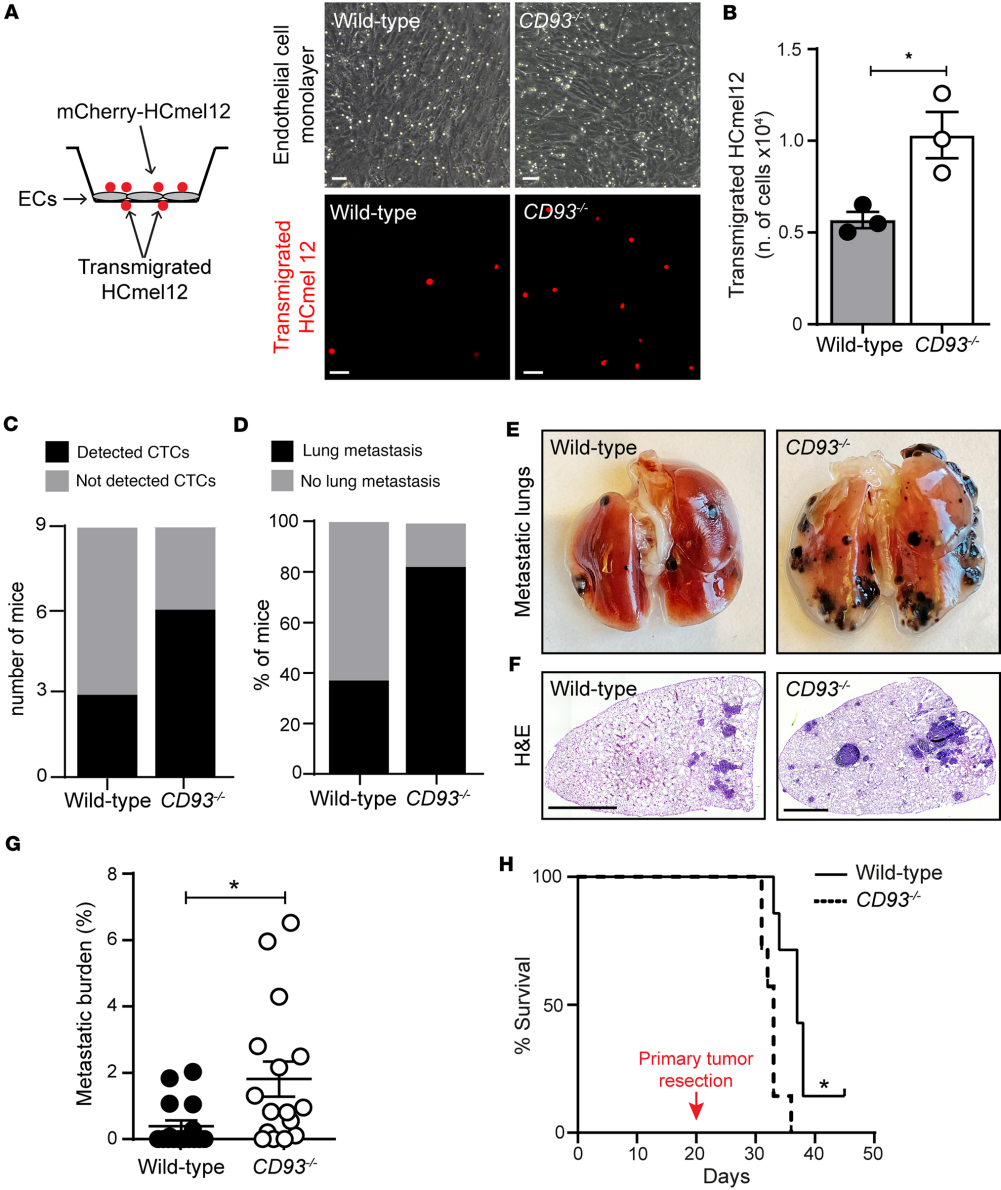
Deletion of CD93 increases endothelial transmigration of tumor cells and promotes metastasis. (**A**) In vitro permeability assay of HCmel12 cells transmigrating through wild-type and *CD93^–/–^* endothelial cell monolayers. Scale bars: 50 μm. (**B**) Graph represents the number of transmigrated tumor cells (3 independent experiments). *P < 0.05 by 2-tailed *t* test. (**C**) Number of wild-type and *CD93^–/–^* mice showing detectable levels of mCherry^+^ circulating tumor cells (CTCs) in the blood 19 days after subcutaneous inoculation of mCherry-HCmel12 cells (*n* = 9/group). *mCherry* expression was detected in the circulation by qPCR. (**D**) Percentage of mice that developed lung metastasis 24 days after subcutaneous inoculation of HCmel12 cells (*n* = 16/group). Representative images of metastatic lungs (**E**) and H&E-stained lung sections (**F**). Scale bars: 1 mm. (**G**) Metastatic burden per mouse (percentage of lung tissue area covered by metastases; lung tilescans of *n* = 16/group). *P < 0.05 by Mann-Whitney test. (**H**) Survival of wild-type and *CD93^–/–^* mice (*n* = 7/group) after tumor resection. *P < 0.05 by Gehan-Breslow-Wilcoxon test. Values represent mean ± SEM.

Next, we assessed whether the compromised endothelial barrier associated with CD93 deficiency can promote transendothelial migration of melanoma cells and formation of distant metastases. For this purpose, we first analyzed the capability of mCherry-HCmel12 cells to transmigrate through a monolayer of endothelial cells isolated from wild-type or *CD93^–/–^* mice grown on transwell inserts (Figure 3A). Notably, *CD93^–/–^* endothelial cells facilitated tumor cell transmigration across the endothelial monolayer, resulting in a significant increase in tumor cells penetrating the endothelial barrier (Figure 3, A and B). In line with this, circulating tumor cells were more frequently detected in the blood of *CD93^–/–^* mice bearing subcutaneous mCherry-HCmel12 tumors as compared with the wild-type group. Indeed, *mCherry* mRNA was detected by qPCR in 6 out of 9 CD93-deficient mice. In contrast, only 3 out of 9 wild-type mice showed detectable levels of *mCherry* mRNA in the blood (Figure 3C and Supplemental Figure 5).

The HCmel12 model spontaneously metastasizes to the lungs when implanted subcutaneously. Therefore, lungs from *CD93^–/–^* and wild-type mice bearing HCmel12 tumors were examined for the presence of metastases 24 days after tumor implantation. Despite the reduced primary tumor growth observed in *CD93^–/–^* mice (Figure 2B), metastatic dissemination to the lungs was increased. Lung metastases were found in 81% of *CD93^–/–^* mice, whereas only 37% of wild-type mice displayed lung metastases (Figure 3D). Moreover, the size of the metastatic lesions in the *CD93^–/–^* mice was significantly increased as compared with the wild-type group (Figure 3, E–G).

To determine whether the increased capacity of melanoma cells to metastasize in *CD93^–/–^* mice would affect their survival after surgical removal of the tumor, primary HCmel12 melanomas were resected on day 20 after subcutaneous injection, when the tumor size was approximately 150 mm^3^. Wild-type and *CD93^–/–^* mice were sacrificed when exhibiting symptoms of lung metastases, such as shortness of breath. The median survival of *CD93^–/–^* mice after resection of the primary tumor was 33 days as compared with 37 days in the wild-type group, consistent with an increased metastatic burden (Figure 3H).

To assess whether CD93 deficiency also affects vascular integrity in other metastatic tumors, we implanted Lewis lung carcinoma cells (LLC1) subcutaneously in wild-type and CD93-deficient mice. Similarly to the HCmel12 model, LLC1 tumor growth was reduced in *CD93^–/–^* mice (Supplemental Figure 6A). The vascular area was not affected by CD93 deficiency in LLC1 tumors, but the levels of VE-cadherin and ZO1 were reduced, and there was a decrease in desmin-positive pericyte coverage, indicating reduced vascular integrity in *CD93^–/–^* mice (Supplemental Figure 6, B–I). Due to the aggressiveness of this tumor model, the absolute majority of wild-type mice and all *CD93^–/–^* mice displayed lung metastasis 14 days after inoculation of LLC1 tumors (Supplemental Figure 6N).

### Endothelial cell–specific deletion of CD93 disrupts vascular integrity and promotes metastases.

**Figure 4:**
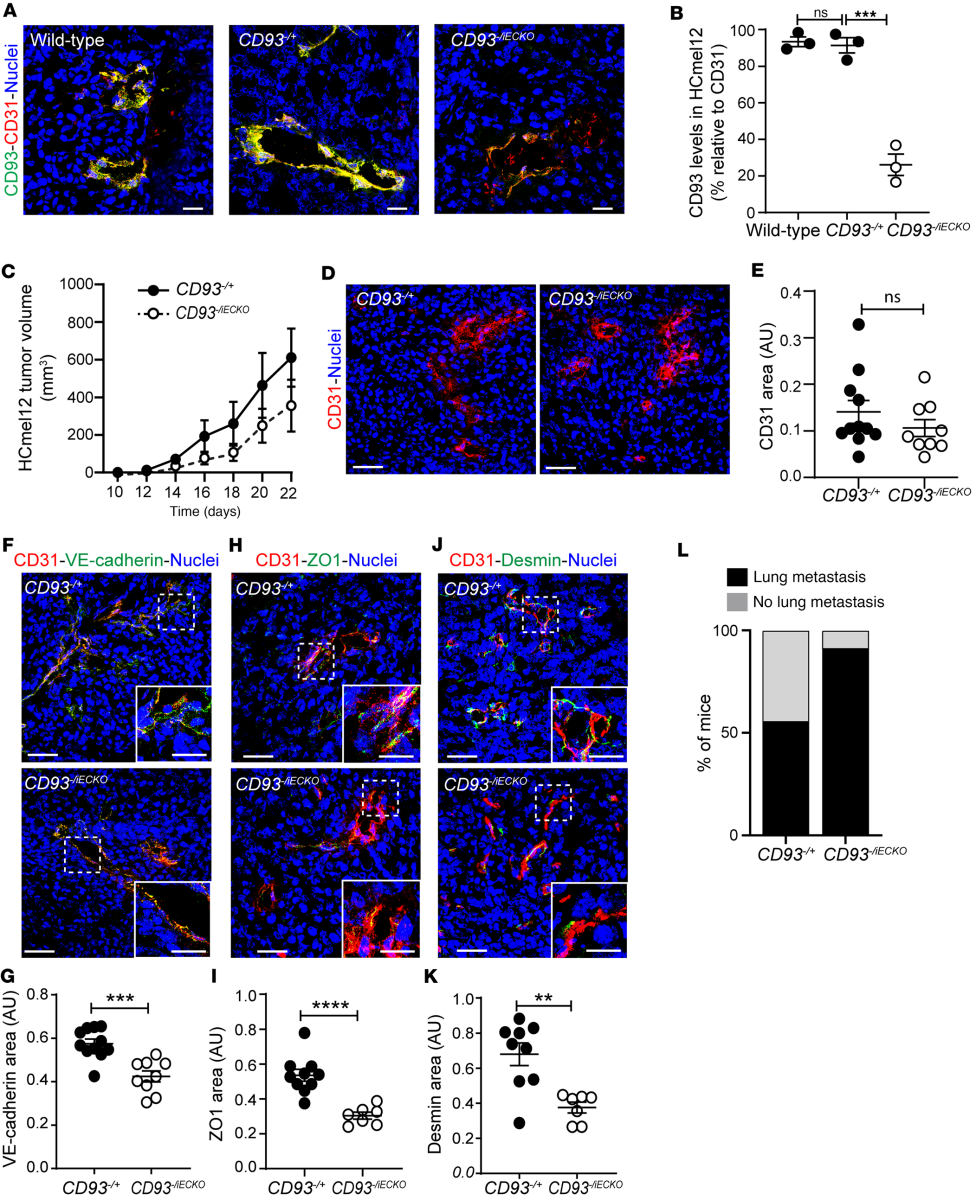
Endothelial cell–specific CD93 deletion impairs HCmel12 vascular integrity and increases metastatic spread. (**A**) Immunofluorescent staining showing CD93 levels (green) in HCmel12 tumors implanted in wild-type, CD93 heterozygous (*CD93^–/+^*), and *CD93^–/iECKO^* mice. Vessels are detected by CD31 (red) and nuclei by Hoechst (blue). Scale bars: 20 μm. (**B**) Quantification of CD93 levels in HCmel12 vessels of wild-type (*n* = 3, 3 fields of view/sample), *CD93^–/+^* (*n* = 3, 3 fields of view/sample), and *CD93^–/iECKO^* (*n* = 3, 3 fields of view/sample) mice. ***P < 0.001 by 1-way ANOVA with Tukey′s multiple-comparison test. NS, not significant. (**C**) Tumor growth in *CD93^–/+^* and *CD93^–/iECKO^* mice (*n* = 11/group). (**D**) Representative images of tumor vessels stained for CD31 (red). Scale bars: 25 μm. (**E**) Quantification of CD31^+^ area in *CD93^–/+^* and *CD93^–/iECKO^* mice (*n* = 11/group, minimum of 4 fields of view/sample). (**F**) Representative images of VE-cadherin (green), (**H**) ZO1 (green), and (**J**) desmin (green) in HCmel12 tumors from in *CD93^–/+^* and *CD93^–/iECKO^* mice. Scale bars: 20 μm and 10 μm (high-magnification insets in **F**, **H**, and **J**). (**G**, **I**, and **K**) Quantification graphs of VE-cadherin, ZO1, and desmin levels normalized by CD31^+^ area. *CD93^–/+^* (*n* = 9, minimum of 4 fields of view/sample), *CD93^–/iECKO^* (*n* = 11, minimum of 4 fields of view/sample). AU, arbitrary units. **P < 0.01, ***P < 0.001, ****P < 0.0001 by 2-tailed *t* test. (**L**) Percentage of mice that developed lung metastasis 22 days after subcutaneous inoculation of HCmel12 cells (*n* = 11/group). Values represent mean ± SEM.

CD93 is mainly expressed in the tumor vessels in murine cancer models, but can also be expressed by some subtypes of hematopoietic cells. To assess whether the disruption of vascular integrity and increased metastatic dissemination observed in *CD93^–/–^* mice is due to the loss of CD93 in endothelial cells, we generated mice with a tamoxifen-inducible endothelial cell–specific deletion of CD93 by crossing *CD93^fl/fl^* mice with Cdh5(PAC)-Cre^ERT2^ mice. Due to the structure of the CD93 gene, the floxed segment was large and led to a mosaic pattern of deletion. Indeed, as shown by the CD93 staining in the retina vasculature of tamoxifen-induced *CD93^fl/fl^* mice, we only observed a minor reduction in CD93 as compared with the control mice (*CD93^fl/fl^* vs. control; Supplemental Figure 7, A and B). Therefore, to increase the proportion of CD93-deleted endothelial cells by only requiring deletion of one allele, we crossed the *CD93^fl/fl^* mice with *CD93^–/–^* mice, and compared heterozygous control mice *CD93^–/+^* mice (Cre-negative *CD93^–/fl^*) with mice with an endothelial cell–specific deletion of CD93 (Cre-positive *CD93*^–/fl^, denoted as *CD93^–/iECKO^*). Tamoxifen treatment in *CD93^–/iECKO^* resulted in an improved *CD93* gene deletion in the retina vasculature and an up to 75% decrease in CD93 expression in HCmel12 tumor vessels in *CD93^–/iECKO^* mice (Supplemental Figure 7, A and B, and Figure 4, A and B, respectively).

Analysis of HCmel12 tumors injected subcutaneously in *CD93^–/iECKO^* mice showed a trend toward decreased tumor growth compared with the control group; however, statistical significance was not reached, likely due to variation in CD93 expression in *CD93^–/iECKO^* mice (Figure 4C). While the vascular area was not affected, the levels of VE-cadherin and ZO1 were reduced, and the desmin-positive pericyte coverage was decreased (Figure 4, D–K). Consistent with reduced vascular integrity in primary HCmel12 tumors in *CD93^–/iECKO^* mice, a higher proportion of these mice displayed metastatic dissemination to the lungs (10 out of 11 mice) as compared with wild-type mice (6 out of 11 mice) (Figure 4L). Together, these results indicate that loss of CD93 in endothelial cells is associated with reduced vascular integrity and enhances metastatic dissemination.

### Increased vascular permeability in the lungs of CD93^–/–^ mice contributes to metastatic spread.

**Figure 5:**
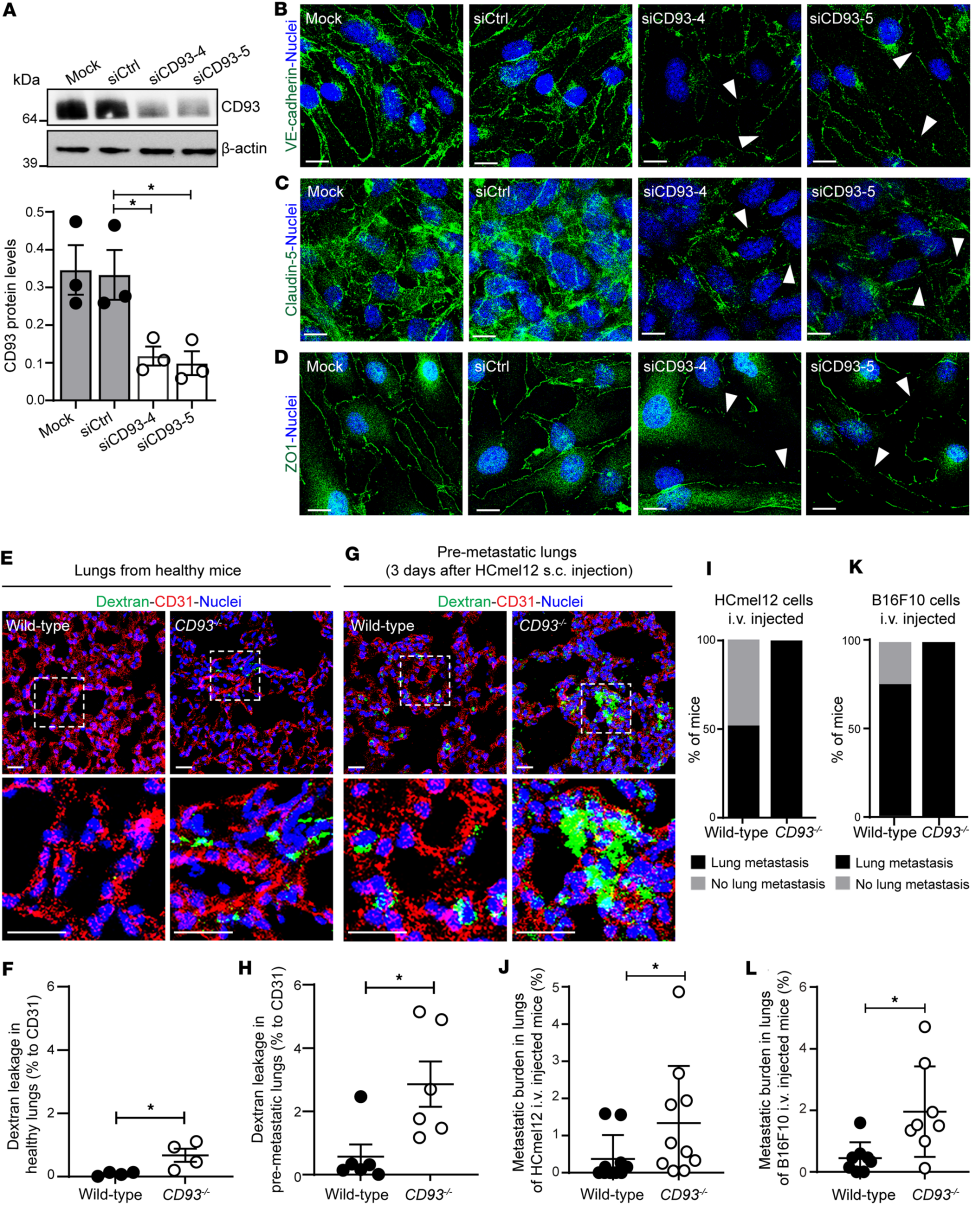
CD93 knockdown impairs endothelial junctions in murine lung endothelial cells and promotes vascular permeability and tumor cell extravasation in lungs. (**A**) Western blot for CD93 and β-actin indicating the silencing efficiency of CD93 siRNAs in the mouse lung endothelial cells (mLECs). Values shown in the graph represent mean ± SEM (3 independent experiments). *P < 0.05 by 1-way ANOVA with Dunnett’s multiple-comparison test. Immunofluorescent staining of VE-cadherin (green) (**B**), claudin5 (green) (**C**), and ZO1 (green) (**D**) in control (Mock and siCtrl) and CD93 siRNA–silenced (siCD93-4 and siCD93-5) mLECs. Scale bars: 20 μm. Arrowheads point toward the disrupted endothelial junctions. Nuclei are visualized by Hoechst staining (blue). Representative images of dextran leakage in healthy (**E**) and in premetastatic lungs (**G**) in wild-type and *CD93^–/–^* mice. Scale bars: 20 μm. (**F** and **H**) Quantification of dextran leakage in wild-type and *CD93^–/–^* mice (*n* = 4–6/group, 4 fields of view/sample). Nuclei are visualized by Hoechst (blue). *P < 0.05 by 2-tailed *t* test. (**I** and **K**) Percentage of mice that developed metastases after intravenous injection (i.v.) of HCmel12 cells (**I**) or B16F10 cells (**K**). (**J** and **L**) Percentage of lung tissue area covered by metastases after i.v. injection of HCmel12 (**J**) and B16F10 cells (**L**). *P < 0.05 by Mann-Whitney test. Values represent mean ± SEM.

To determine whether CD93 regulates the endothelial barrier function in the lungs, which is a major site of metastatic dissemination, we first assessed the effect of CD93 silencing on endothelial cell-cell junctions and ECM deposition in vitro in murine lung endothelial cells (mLECs) (Figure 5, A–D). Notably, CD93 downregulation (Figure 5A) was associated with a disruption of the endothelial junction proteins VE-cadherin, claudin-5, and ZO1 (Figure 5, B–D, respectively), indicating an important role of CD93 in the stability of the cell-cell junctions in lung endothelial cells. To further explore to what extent CD93 deficiency regulates vessel permeability in lungs, 70 kDa biotinylated dextran was administered intravenously into healthy wild-type and *CD93^–/–^* mice, followed by a 6-hour circulation period. In accordance with the disruption of the endothelial junctions observed in vitro, a weak but significant accumulation of extravasated dextran was found in the lungs of *CD93^–/–^* mice compared with the minimal signal detected in the lungs of wild-type mice (Figure 5, E and F). Interestingly, dextran leakage was further increased in lungs isolated from CD93-deficient mice subcutaneously injected with HCmel12 after 3 days from the tumor inoculation (Figure 5, G and H), indicating that CD93 deficiency promotes the formation of a premetastatic niche in the lungs.

Finally, to assess whether tumor cell extravasation and seeding in lung tissue were facilitated by CD93 deficiency, melanoma cells were injected via the tail vein into the circulation of wild-type and *CD93^–/–^* mice. Lung metastases were observed in all mice in the *CD93^–/–^* group 24 days after HCmel12 injection, while only 50% of the wild-type mice displayed metastases (Figure 5I). The metastatic burden, as assessed by the area of lung tissue covered by HCmel12 metastases, was increased in *CD93^–/–^* mice as compared with the wild-type group (Figure 5J). Similarly, intravenous injection of B16F10 cells resulted in formation of lung metastases in all injected *CD93^–/–^* mice as compared with 75% of mice in the wild-type group (Figure 5K), and the metastatic burden was significantly increased in CD93-deficient mice (Figure 5L).

Taken together, these data indicate that CD93 deficiency increases vascular permeability in the lungs and contributes to the formation of a premetastatic niche that facilitates extravasation of circulating tumor cells and metastasis formation.

### CD93 limits the activation of VEGFR2 by promoting its interaction with VE-PTP.

VEGFR signaling is well known to be involved in the dissemination of metastatic cells, and CD93 interacts with several proteins that have been implicated in modulating the VEGF signaling pathway (18–20).

**Figure 6:**
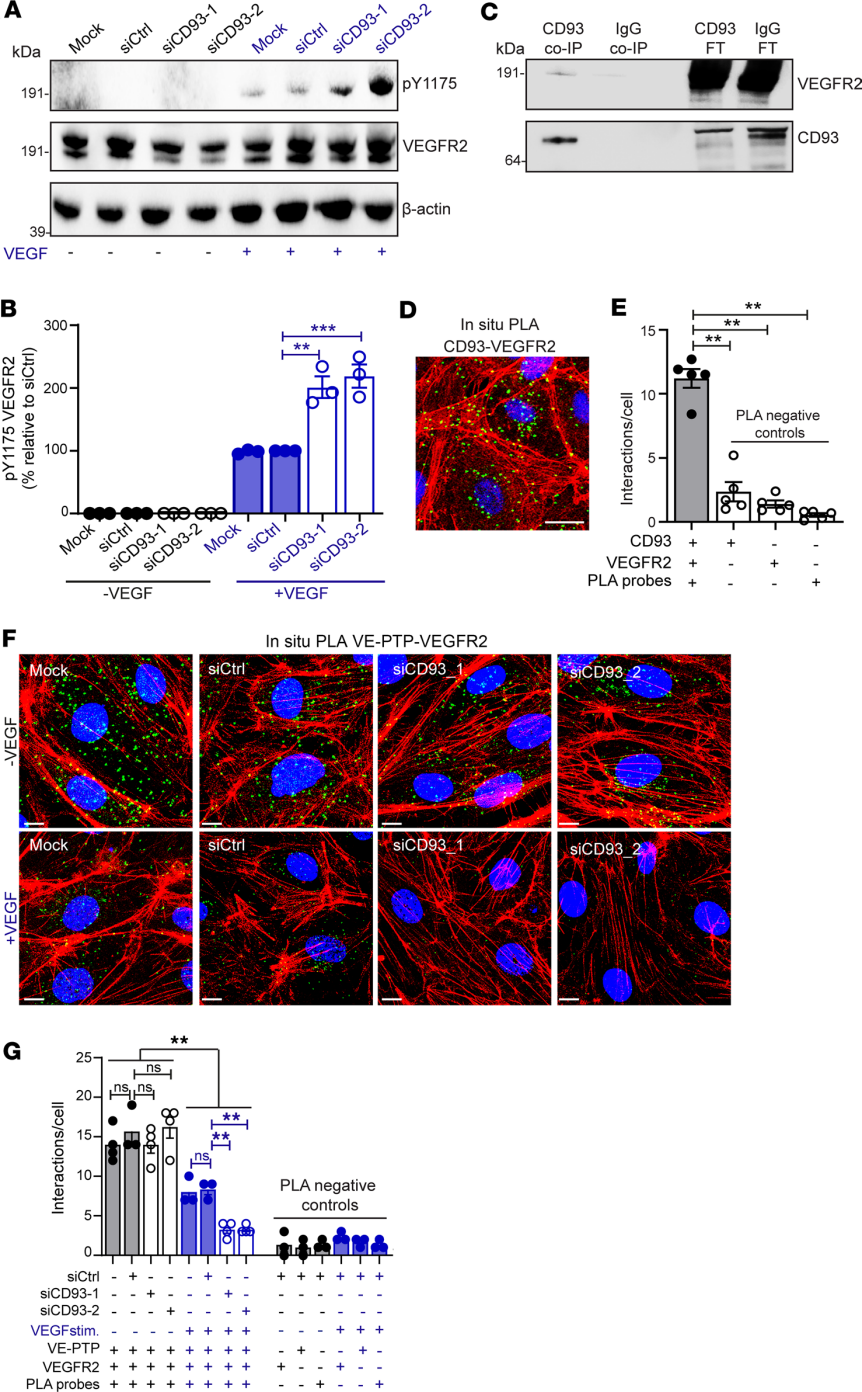
CD93 interacts with VEGFR2 and attenuates its phosphorylation in response to VEGF by promoting VE-PTP–VEGFR2 interaction. (**A**) Western blot to detect p-Y1175 VEGFR2, total VEGFR2, and β-actin in HDBECs stimulated with/without VEGF (10 ng/mL, 5 minutes). (**B**) Quantification of p-Y1175 VEGFR2 normalized to the total VEGFR2 (3 independent experiments). (**C**) Western blot to detect VEGFR2 in CD93 and IgG coimmunoprecipitated samples (CD93 Co-IP and IgG Co-IP) and flow-through samples (CD93 FT and IgG FT) derived from HDBEC protein lysates. (**D**) In situ PLA for CD93 and VEGFR2 in HDBECs. Scale bar: 25 μm. (**E**) Quantification of CD93-VEGFR2 interaction (green dots) relative to cell number (5 fields of view/sample). (**F**) In situ PLA for VE-PTP and VEGFR2 in HDBECs with/without VEGF (10 ng/mL, 5 minutes). Scale bars: 25 μm. (**G**) Quantification of VE-PTP–VEGFR2 interactions relative to cell number (4 fields of view/sample). **P ≤ 0.01; ***P < 0.001 by 1-way ANOVA with Dunnett’s multiple-comparison test. NS, not significant. Values represent mean ± SEM.

To investigate the molecular mechanism through which CD93 affects metastatic dissemination, we first determined whether CD93 participates in crosstalk with the VEGF signaling pathway. Notably, siRNA-mediated knockdown of CD93 resulted in a significant increase in phosphorylation of VEGFR2 on Y1175 upon VEGF stimulation in human dermal blood endothelial cells (HDBECs), as compared with control conditions (Figure 6, A and B). Moreover, coimmunoprecipitation (co-IP) using HDBEC protein extracts revealed a direct binding between CD93 and VEGFR2 (Figure 6C). This interaction was further validated by in situ proximity ligation assay (PLA) in human endothelial cell monolayers, indicating formation of CD93-VEGFR2 complexes (Figure 6, D and E).

To further explore the mechanisms involved in CD93 regulation of VEGFR2 activation, we analyzed the interactions between VEGFR2 and VE-PTP in CD93 siRNA–treated endothelial cells and controls using PLA. VE-PTP–VEGFR2 interactions were readily detected and their prevalence did not differ between unstimulated control and CD93 siRNA–treated cells (“–VEGF” in Figure 6, F and G). Upon VEGF stimulation, VE-PTP–VEGFR2 interactions significantly decreased in control endothelial cells (“+VEGF” in Figure 6, F and G). Notably, the destabilization of this complex in response to VEGF was further enhanced in CD93-silenced cells. Indeed, the number of positive interactions per cell detected in CD93-downregulated cells stimulated with VEGF was significantly reduced as compared with the control condition under VEGF stimuli (graph in Figure 6G). In contrast, downregulation of VE-PTP did not affect complex formation between VEGFR2 and CD93 (Supplemental Figure 8, A–C).

To investigate the role of VE-PTP in disruption of endothelial junctions, we performed single or double knockdown of CD93 and VE-PTP in mLECs. Knockdown of VE-PTP reduced VE-cadherin in endothelial junctions, consistent with a role in regulating VEGFR2 signaling, but did not alter the level of ZO1 in endothelial junctions (Supplemental Figure 9, A–E). Notably, VE-PTP knockdown did not further reduce VE-cadherin expression in CD93-silenced endothelial cells, supporting the notion that silencing of CD93 prevents dephosphorylation of VEGFR2 through VE-PTP (Supplemental Figure 9, B and C).

Together, these data indicate that CD93 promotes the interaction between VE-PTP and VEGFR2, thereby limiting VEGF-induced phosphorylation and activation of VEGFR2 signaling.

### CD93 deletion induces overexpression of MMP9 in response to VEGF.

**Figure 7:**
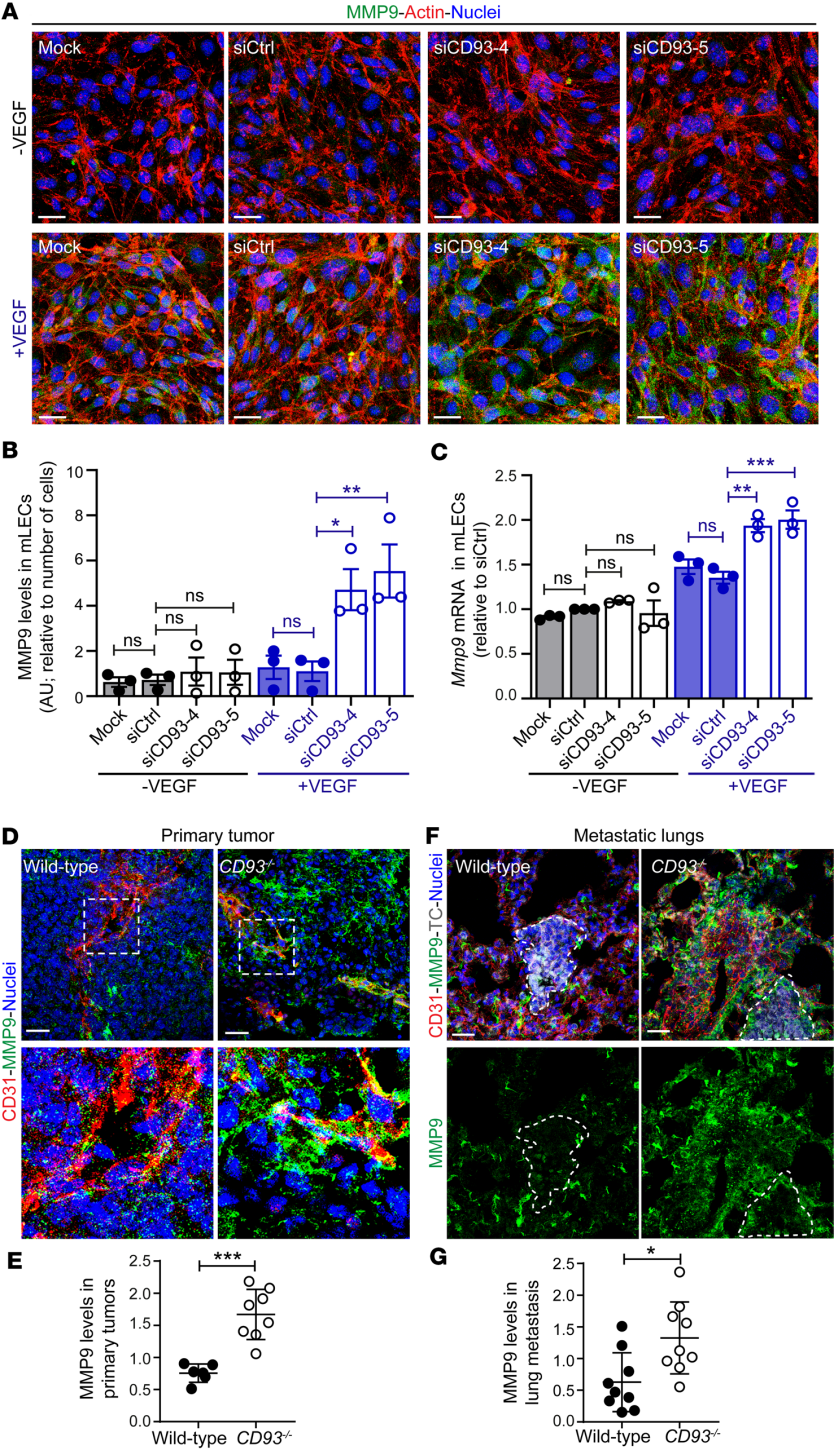
CD93 regulates MMP9 levels in vitro as well as in primary and metastatic sites. (**A**) Immunofluorescence images of MMP9 (green) in control mLECs (Mock and siCtrl) and mLECs silenced for CD93 (siCD93-4 and siCD93-5) with/without VEGF (10 ng/mL, 5 minutes). Actin and nuclei were visualized by phalloidin (red) and Hoechst (blue). Scale bars: 20 μm. (**B**). Quantification of MMP9 levels in mLECs. *P < 0.05, **P < 0.01 by 1-way ANOVA with Tukey′s multiple-comparison test (3 independent experiments). (**C**) Real-time qPCR showing *Mmp9* mRNA levels in control mLECs (mock and siCtrl) and CD93-silenced mLECs (siCD93-4 and siCD93-5) with/without VEGF (3 independent experiments). **P < 0.01, ***P < 0.001 by 1-way ANOVA with Tukey′s multiple-comparison test. (**D**) Immunofluorescent staining of MMP9 (green) and CD31 (red) in HCmel12 primary tumor from wild-type and *CD93^–/–^* mice. Scale bars: 20 μm. (**E**) Quantification of the area covered by MMP9 normalized to the CD31^+^ area in wild-type (*n* = 5) and *CD93^–/–^* (*n* = 7) HCmel12 primary tumor tissue. (**F**) Immunofluorescent staining of MMP9 (green) and CD31 (red) in metastatic lungs from wild-type and *CD93^–/–^* mice. Metastatic lesion of mCherry-HCmel12 tumor cells (TC, gray) are defined by dotted line. Scale bars: 20 μm. (**G**) Quantification of the area covered by MMP9 around the lung metastatic lesion normalized to the CD31^+^ area in wild-type (*n* = 9) and *CD93^–/–^* (*n* = 9) lung metastatic lesions. All immunofluorescence quantifications were performed in a minimum of 4 fields of view/sample. AU, arbitrary units. *P < 0.05; **P < 0.01; ***P < 0.001 by 2-tailed *t* test (**E** and **G**). Values represent mean ± SEM.

VEGFR2 activation enhances endothelial MMP9 expression in the tumor microenvironment, which degrades the ECM and facilitates metastatic dissemination (29–31). To determine whether the increased VEGFR2 activation observed in the absence of CD93 was associated with elevated levels of MMP9, we silenced CD93 in mLECs and analyzed the MMP9 signal. Consistent with an enhanced response to VEGF upon CD93 knockdown, we observed a significant upregulation of MMP9 levels in VEGF-stimulated siCD93-treated mLECs compared with VEGF-treated control mLECs (Figure 7, A and B). *Mmp9* mRNA levels were significantly increased after VEGF stimulation in CD93-silenced cells as compared with control, consistent with MMP9 being transcriptionally regulated downstream of VEGFR2 activation (Figure 7C). Knockdown of VE-PTP increased MMP9 expression, but did not further enhance MMP9 levels in CD93-silenced cells, supporting the notion that CD93 regulates MMP9 through VE-PTP (Supplemental Figure 9, F and G).

MMPs are upregulated in the tumor tissues and promote tissue colonization by metastatic cells (30, 31); therefore, we asked whether the increased incidence of lung metastases observed in *CD93^–/–^* mice was associated with MMP9 upregulation. For this purpose, we first analyzed MMP9 levels in subcutaneous HCmel12 tumors and metastatic lungs. A substantial increase in MMP9 expression was detected in the perivascular region of HCmel12 tumors in *CD93^–/–^* mice as compared with those in wild-type mice (Figure 7, D and E). A similar increase in MMP9 expression was noted in B16F10 melanomas and LLC1 tumors (Supplemental Figure 4, I and J, and Supplemental Figure 6, J and K). Moreover, while MMP9 was expressed at low levels in the normal lung tissues in both groups (Supplemental Figure 10, A and B), a significant upregulation was observed in close proximity to the metastatic lesions in the lungs of *CD93^–/–^* mice (Figure 7, F and G).

**Figure 8:**
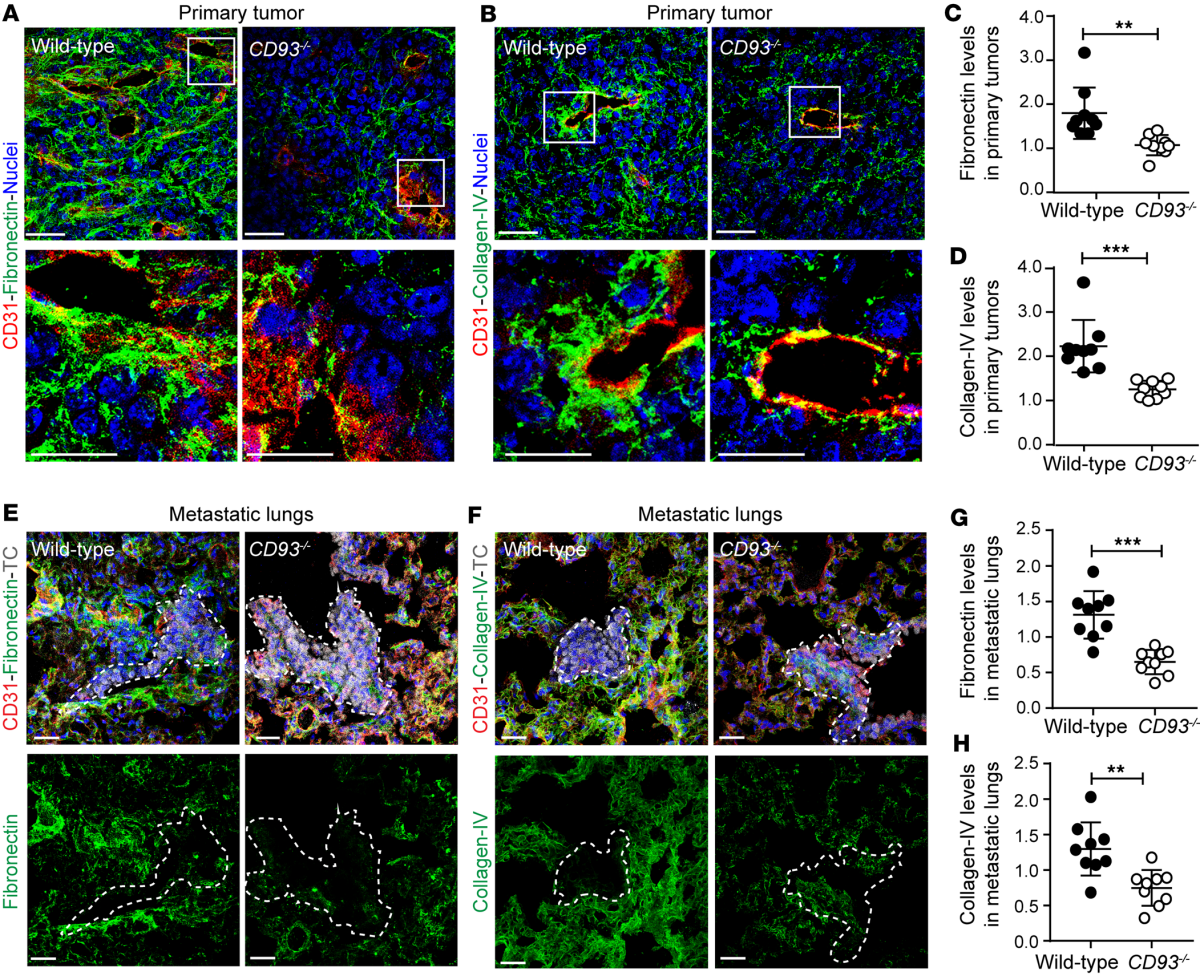
CD93 deficiency impairs extracellular matrix deposition in the primary tumor site and at the metastatic lungs. Representative immunofluorescent staining of fibronectin (green) (**A**) and collagen IV (green) (**B**), in the primary HCmel12 tumor vasculature of wild-type and *CD93^–/–^* mice. Vasculature is detected by CD31 staining (red). Nuclei are visualized by Hoechst (blue). (**C** and **D**) Quantification of fibronectin^+^ and collagen IV^+^ area in primary HCmel12 tumor vasculature (*n* = 9/group, minimum of 6 fields of view/sample). **P < 0.01; ***P < 0.001 by 2-tailed *t* test. (**E** and **F**) Representative immunofluorescence images of fibronectin (green) and collagen IV (green) in the metastatic lungs of wild-type and *CD93^–/–^* mice. Metastatic lesions of mCherry-HCmel12 tumor cells (TC, gray) are defined by a dotted line and the vasculature is detected by CD31 staining (red). Nuclei are visualized by Hoechst (blue). (**G** and **H**) Quantification of fibronectin^+^ and collagen IV^+^ area around the metastatic lesions in wild-type and *CD93^–/–^* lungs (*n* = 9/group, minimum of 3 fields of view/sample). Scale bars: 20 μm and 10 μm (high-magnification insets in **A** and **B**). AU, arbitrary units. **P < 0.01; ***P < 0.001 by 2-tailed *t* test. Values represent mean ± SEM.

MMPs are key regulators of ECM remodeling, promoting cell invasion and metastatic growth (32). In line with the increased MMP9 expression around the tumor vasculature, a substantial reduction in the deposition of the ECM proteins fibronectin and collagen IV was observed in the perivascular region of HCmel12 melanomas in *CD93^–/–^* mice (Figure 8, A–D). Consistent with this, fibronectin levels were also reduced in the perivascular regions of B16F10 and LLC1 tumors in *CD93^–/–^* mice (Supplemental Figure 4, K and L, and Supplemental Figure 6, L and M). The amount of fibronectin and collagen IV in healthy lungs was not affected by CD93 deficiency (Supplemental Figure 10, C–F). However, a strong reduction in fibronectin and collagen IV was observed in proximity of the HCmel12 metastatic lesions in *CD93^–/–^* mice (Figure 8, E–H). Taken together, the data indicate that hyperactivation of VEGFR2 in response to CD93 deletion leads to enhanced MMP9 levels and degradation of ECM in the metastatic lesions.

### Inhibition of VEGFR2 in CD93-deficient mice restores endothelial barrier function and decreases metastatic dissemination.

Next, we addressed whether the pronounced destabilization of the tumor vasculature and the increased metastasis formation observed in *CD93^–/–^* mice was dependent on the hyperactivation of VEGFR2. For this purpose, wild-type and *CD93^–/–^* mice bearing subcutaneous HCmel12 tumors were treated with the VEGFR2-blocking antibody DC101 or the corresponding isotype control.

**Figure 9:**
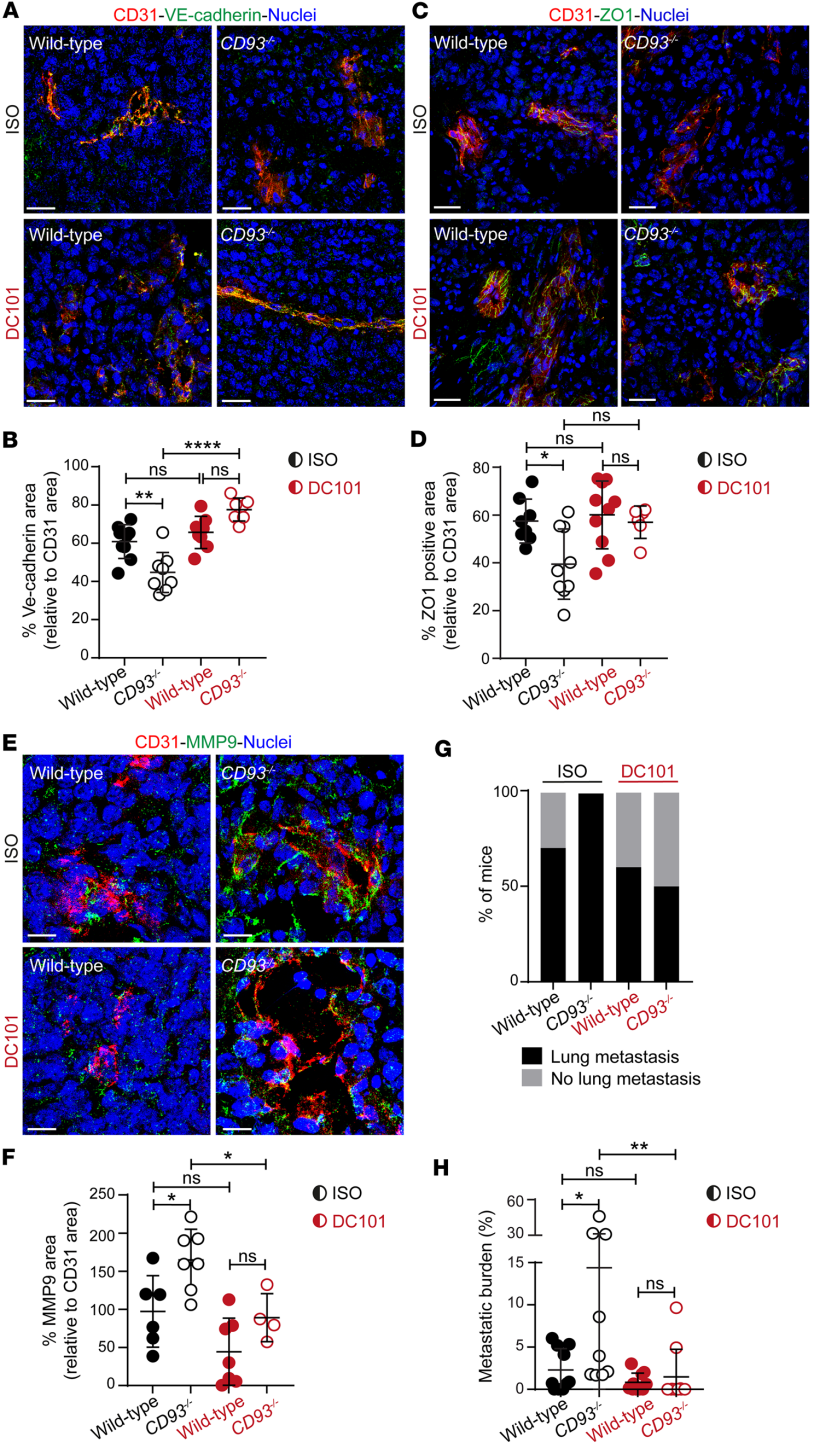
VEGFR2 inhibition restores tumor vessel integrity in CD93-deficient mice and reduces metastatic dissemination. Immunofluorescent staining of VE-cadherin (**A**), ZO1 (**C**), and MMP9 (**E**) in HCmel12 subcutaneous tumors from wild-type and *CD93^–/–^* mice treated with DC101 or isotype control (ISO). Scale bars: 20 μm. (**B**, **D**, and **F**) VE-cadherin (**B**), ZO1 (**D**), and MMP9 (**F**) expression was quantified and normalized to the CD31^+^ area. Wild-type ISO (*n* = 6–8), *CD93^–/–^* ISO (*n* = 7–9), wild-type DC101 (*n* = 7–10), and *CD93^–/–^* DC101 (*n* = 4–6). *P < 0.05; **P < 0.01; ****P < 0.0001 by 1-way ANOVA with Tukey’s multiple-comparison test. NS, not significant. All immunofluorescence quantifications were performed in a minimum of 5 fields of view/sample**.** (**G**) Percentage of mice that developed lung metastasis after subcutaneous inoculation of HCmel12 cells. Wild-type ISO (*n* = 10), *CD93^–/–^* ISO (*n* = 9), wild-type DC101 (*n* = 10), and *CD93^–/–^* DC101 (*n* = 10). (**H**) Metastatic burden per mouse (percentage of lung tissue area covered by metastases). Wild-type ISO (*n* = 10), *CD93^–/–^* ISO (*n* = 9), wild-type DC101 (*n* = 10), and *CD93^–/–^* DC101 (*n* = 10). *P < 0.05, **P ≤ 0.01 by 1-way ANOVA with Tukey’s multiple-comparison test.

Interestingly, the expression levels of the adherens and tight junctional molecules VE-cadherin and ZO1, respectively, as well as the MMP9 expression observed in isotype-treated *CD93^–/–^* mice were restored to wild-type levels upon DC101 treatment (Figure 9, A–F).

We next assessed whether the improved tumor endothelial barrier function observed in response to VEGRF2 inhibition in *CD93^–/–^* mice would reduce the metastatic spread. Lung metastases were observed in all isotype-treated *CD93^–/–^* mice and in 75% of isotype-treated wild-type mice (Figure 9G). Strikingly, after DC101 therapy, the proportion of *CD93^–/–^* mice with lung metastases was reduced (Figure 9G) and the metastatic burden was decreased to the levels observed in the wild-type mice (Figure 9H).

Collectively, our data demonstrate that CD93 deficiency results in metastatic dissemination by impairing the vessel integrity in a VEGFR2-dependent manner.

## Discussion

CD93 has emerged as a potential target for antiangiogenic therapy, but its impact on vascular stability and metastatic dissemination has not been evaluated prior to this study. Metastasis formation is a multistep process that involves cancer cell transmigration through tumor vessels during intravasation and through the healthy vasculature of the homing organ during extravasation (33). The vascular barrier formed by endothelial cells, ECM, and pericytes limits metastatic dissemination (4, 34, 35). Here, we demonstrate that CD93 deficiency destabilizes the primary tumor vasculature, facilitating the intravasation of tumor cells, and creates a permissive microenvironment at the metastatic site. The effects on vascular integrity observed in CD93-deficient mice were dependent on VEGFR2 signaling, as treatment with DC101 restored the endothelial barrier function and reduced metastatic dissemination to the level of wild-type controls. Consistent with our data, genetic deletion of MMRN2 in mice leads to disengagement of endothelial junctions and decreased pericyte coverage, which are associated with elevated VEGFR2 signaling (36). This indicates that the CD93-MMRN2 complex plays a crucial role in regulating tumor vessel stability through modulating the response of VEGFR2 to VEGF stimulation. Conversely, treating tumor-bearing mice with a neutralizing antibody against CD93 that blocks its interaction with IGFBP7 was associated with vascular normalization (14). This may be explained by the fact that, unlike the total gene deletion in *CD93^–/–^* mice, the anti-CD93 antibody does not block all functions of CD93. CD93 can interact with many different molecular partners and the antibody used by Sun et al. (14) blocks the binding between CD93 and IGFBP7, which may not be a crucial interaction for the regulation of endothelial cell junction stability. This indicates that the net impact of CD93 on vascular phenotype strictly depends on its molecular interactions with other proteins. CD93 is phosphorylated in endothelial cells, which is necessary for endothelial migration and creates a docking site for the interaction with CBL, which affects Rho GTPase signaling and endothelial junctions (27, 37, 38). Our data indicate that CD93 participates in molecular networks that stabilize tumor vessels, including those that regulate VEGFR2 activation.

Notably, CD93 deficiency in endothelial cells was associated with an increased phosphorylation of VEGFR2 after VEGF stimulation. VEGFR2 phosphorylation is increased in the absence of VE-PTP in endothelial cells (8). VE-PTP acts via the angiopoietin receptor TIE2 to maintain VEGFR2 in a dephosphorylated state and inhibits its activation (9, 39). Interestingly, CD93 was recently identified as a putative substrate of VE-PTP in a substrate trapping experiment (21). Here, we demonstrate that CD93 binds to VEGFR2 and promotes its interaction with VE-PTP, suggesting a potential mechanism through which CD93 mitigates VEGFR2 activation and signaling. Alternatively, CD93 may modulate the activation of VEGFR2 by modifying its 3-dimensional structure or its endocytosis (40). In addition, CD93 binds to CBLC, a ubiquitin protein ligase that can inhibit VEGF/VEGFR2–driven angiogenesis by suppressing PLCγ activation, which may be an additional pathway of regulation (38, 41). CD93 is a part of the group XIV C-type lectin superfamily, which also includes endosialin, thrombomodulin, and CLEC14A (42–44). CD93 and CLEC14A have overlapping binding sites in MMRN2, and appear to elicit similar responses in tumor vasculature upon deficiency (15, 16, 45). Indeed, deletion of CLEC14A in mice results in elevated VEGFR2 signaling and increases tumor vessel leakage (45, 46). It has been suggested that MMRN2 sequesters VEGFA and that its absence therefore enhances VEGFR2 signaling within the tumors (19, 36). However, whereas loss of CLEC14A modulates MMRN2 expression, CD93 does not (15, 45). Therefore, enhanced VEGFR2 signaling associated with CD93 deficiency is not secondary to increased availability of VEGF due to loss of MMRN2. This notion is consistent with enhanced VEGFR2 signaling following CD93 knockdown in endothelial cells observed in vitro, where there is no shortage of available ligand.

VEGF/VEGFR2 signaling promotes metastatic spread to secondary organs by inducing vessel permeability (4), facilitating invasion of tumor cells into metastatic tissues through augmenting expression of MMPs such as MMP9 that degrade the ECM (5). In line with a hyperresponsiveness to VEGF, we observed a substantial increase in MMP9 levels in CD93-deficient subcutaneous tumors and lung metastases, which was associated with enhanced invasion and improved colonization of tumor cells. MMP9 is also expressed by tumor-associated macrophages and neutrophils (47), which may contribute to MMP9 expression in primary tumors and metastases. Nevertheless, our data suggest that the increased responsiveness of tumor endothelial cells to VEGF underlies the high MMP9 expression in CD93-deficient tumors. MMP9 can regulate ECM remodeling by proteolytic cleavage, including degradation of collagen IV (48). CD93, on the other hand, is necessary for integrin β1 activation and fibronectin fibrillogenesis (15). Consistent with this, collagen IV and fibronectin were reduced in perivascular regions in primary melanomas and metastasis in *CD93^–/–^* mice.

Our results demonstrate that decreased vascular integrity in *CD93^–/–^* tumors is largely dependent on enhanced VEGFR2 signaling, and underscore that vascular permeability has a major impact on metastatic spread. Taken together, our data identify CD93 as a key regulator of endothelial barrier function in tumors through modulating VEGFR2 function, which has important implications for its therapeutic targeting in cancer.

## Methods

Additional and detailed materials and methods as well as reagent specifications are provided in the Supplemental Material.

### Sex as a biological variable.

Both males and females were evaluated in the study. There were no differences related to sex with regard to the parameters we measured.

### TMA.

Expression of CD93, MMRN2, and fibronectin in tumor vessels was analyzed in TMAs containing duplicate tissue cores per sample from primary lung cancer (*n* = 60), lung metastatic lesions (*n* = 50), and melanoma metastatic lesions (*n* = 20). Among the analyzed samples, 15 patient-matched sample pairs from primary and metastatic lung lesions were included (23). TMA cores were semiquantitatively scored by 2 researchers in a blinded fashion on a scale of 0 to 2 (0 = no vessel staining, 1 = medium intensity, and 2 = high intensity). Immunohistochemical staining was performed as previously described (49).

### Cells.

Parental and mCherry-tagged HCmel12 melanoma cells were provided by Thomas Tüting (Laboratory of Experimental Dermatology, University of Bonn, Bonn, Germany). B16F10 melanoma cells (CRL-6475) and LL/2 (LLC1) Lewis lung carcinoma cells (CRL-1642) were obtained from ATCC. HDBECs (C-12211) were obtained Promocell. Primary mouse endothelial cells were isolated from wild-type and *CD93^–/–^* mice as described in Paolinelli et al. (50) and mLECs were generated as described in Bussolino et al. (51). All cells were routinely tested for mycoplasma.

### Mice.

*CD93^–/–^* mice (52) and wild-type (*CD93^+/+^*) littermates were bred in house. C57BL/6 mice were purchased from Taconic Biosciences. CD93 conditional knockout (*CD93^fl/fl^*) mice were generated by Taconic Biosciences through the insertion of 2 *loxP* sequences flanking exons 1 and 2 of the murine *CD93* gene to produce a gene deletion after Cre-mediated recombination. The CD93 endothelial cell–specific knockout mice were generated by crossing *CD93^fl/fl^* mice with Cdh5(PAC)-Cre^ERT2^ mice (donated by Ralf H. Adams, University of Munster, Muenster, Germany) (53). Cre-mediated recombination was induced by 5 consecutive intraperitoneal injections of 1 mg of tamoxifen (T5648, Sigma-Aldrich) dissolved in corn oil. Mice were allowed to rest for 5 days before tumor implantation. Due to a poor CD93 gene deletion, characterized by a mosaic pattern of deletion in blood vessels, *CD93^fl/fl^*-Cdh5(PAC)-Cre^ERT2^ mice were bred with constitutive CD93-knockout mice (*CD93^–/–^*), generating a mouse strain characterized by one CD93 allele constitutively deleted and the other allele specifically knocked out in endothelial cells after Cre-mediated recombination (*CD93^–/fl^*, denoted as *CD93*^–/iECKO)^. Endothelial cell–specific CD93 deletion was tested after tamoxifen induction in retinal vasculature of adult mice. *CD93^–/iECKO^* mice showed reduced CD93 levels as compared with *CD93^fl/fl^* (Supplemental Figure 7, A and B). CD93 protein levels in vessels of *CD93^–/+^* were comparable to the levels observed in the vessels of wild-type mice (Supplemental Figure 7, A and B) and CD93 heterozygous mice do not display any vessel phenotype due to the deletion of one allele (15).

### Tumor studies.

Tumor growth and spontaneous lung metastases formation was analyzed in 9- to 12-week-old wild-type and *CD93^–/–^* mice as well as in *CD93^–/iECKO^* subcutaneously injected with 2 × 10^5^ parental HCmel12 or mCherry-HCmel12 tumor cells. B16F10 tumor cells (2.5 × 10^5^ cells) as well as LLC1 (5 × 10^5^ cells) were subcutaneously injected in wild-type and *CD93^–/–^* mice. Tumors were measured by caliper, and the tumor volume was calculated according to the following formula: *V* = 4/3π × length × width × depth. Twenty-four days after the HCmel12 injection or 18 days after the B16F10 or 14 days after LLC1 injection, mice were sacrificed and tumors and lungs were collected for analysis.

For the survival study, HCmel12 cells were inoculated subcutaneously in wild-type and *CD93^–/–^* mice. Twenty days after the tumor cell inoculation, primary tumors were surgically resected. Mice were sacrificed when they exhibited signs of respiratory distress or decreased body weight.

To investigate metastatic seeding of tumor cells into the lungs, mice were intravenously injected with 7.5 × 10^5^ HCmel12 cells or 5 × 10^5^ B16F10 cells in the tail vein. Lungs were harvested 24 days after tumor cell injection and processed for histological analysis.

To investigate the role of VEGFR2 activation in metastatic dissemination, a total of 4 treatments of 400 μg of monoclonal antibody DC101 (BE0060, BioSite) or its isotype control rIgG2a (BE0088, BioSite), were injected intraperitoneally every third day starting from the observation of palpable tumor. Mice were sacrificed 2 days after the last treatment and tumors and lungs were collected for analysis.

Tumor hypoxia was assessed by intraperitoneal injection of Hypoxyprobe Red549 (HP7-x, Hypoxyprobe Inc) 1 hour prior to sacrificing the mice, enabling detection of hypoxic areas in cryosections by immunostaining using the Dylight-549–labeled fluorescent antibody.

### In vivo lung permeability assay.

Biotinylated dextran 70 kDa (D1957, Thermo Fisher Scientific) was injected into the tail vein of wild-type and *CD93^–/–^* mice and allowed to circulate for 6 hours. Mice were anesthetized and perfused with 1× PBS followed by 4% paraformaldehyde through the pulmonary circulation. Lungs were harvested and stored for further analysis.

### Circulating tumor cell analysis.

Blood was collected from wild-type and *CD93^–/–^* mice by cardiac puncture 19 days after subcutaneous inoculation of mCherry-HCmel12 cells. A total volume of 300 μL of blood was immediately mixed with anticoagulant (ACD buffer; 38 mmol/L citric acid, 75 mmol/L trisodium citrate, and 100 mmol/L dextrose) followed by RNA extraction by using the RiboPure Blood Kit (Life Technologies/Ambicon), according to the manufacturer’s instructions.

### RNA extraction and quantitative PCR.

RNA isolated from blood or HCmel12 tumor tissues of wild-type and *CD93^–/–^* mice was extracted using the RNeasy Plus Mini Kit (Qiagen).

Total RNA was transcribed using an iScript cDNA synthesis Kit (1708891, Bio-Rad) and mRNA expression of *mCherry* was quantified relative to *HPRT* by real-time qPCR, in duplicate reactions per sample, with 0.25 μM forward and reverse primers in SYBR Green PCR master mix (Life Technologies). Primer sequences are listed in Supplemental Methods.

### Immunofluorescent staining of tumor and lung sections.

For immunofluorescent staining, primary tumors or lungs were embedded in OCT. Cryosections were cut into 10-μm sections and fixed in ice-cold acetone for 15 minutes. Sections were blocked in PBS containing 3% bovine serum albumin for 1 hour at room temperature followed by overnight incubations with specific primary antibodies. Sections were subsequently incubated with Alexa Fluor–conjugated secondary antibodies (Invitrogen) for 1 hour at room temperature. Images were obtained using a confocal microscope (Leica SP8) or a fluorescence microscope (Leica DMi8).

### Co-IP.

Co-IP for CD93 was performed as described previously (15) in total protein extracts obtained from cultured HDBECs by using a Pierce co-IP kit (26149, Thermo Fisher Scientific).

### PLA.

PLA was performed on HDBECs using the Duolink kit (DUO92001, Sigma-Aldrich) according to the manufacturer’s protocol. Anti-VEGFR2 (2479S, Cell Signaling Technology), anti–VE-PTP (sc-1114, Santa Cruz Biotechnology), and anti-CD93 (D198-3, MBL Life Science) antibodies as well as specific PLA secondary probes were used. At least 5 different areas of the cell monolayer were analyzed.

### Image analysis.

Image analysis was performed by using ImageJ software v.1.51 (NIH). Quantification of vessel-associated markers in tumor samples was performed on a predetermined perivascular region (ROI = 20 pixels from the abluminal vessel side). All immunofluorescence quantifications were performed on a minimum of 3 fields of view/sample. Analysis of fibrinogen and dextran leakage, Glut1 and hypoxyprobe, as well as the analysis of metastatic burden was performed on tilescans of the entire tissue section. Stereological quantification of vessel parameters was performed as previously described (54).

### Statistics.

Statistical analysis was performed using GraphPad Prism 7.0 software. Statistical differences between groups were analyzed using 2-tailed *t* test, Mann-Whitney test, 1-way or 2-way ANOVA test, as specified in the figure legends. A *P* value of less than or equal to 0.05 was considered statistically significant.

### Study approval.

Human tissue was obtained in a manner compliant with the Declaration of Helsinki. The Ethics Review Board in Uppsala approved the use of human samples (Dnr.2010/291, Dnr.2007/159). Participation of patients occurred after informed consent. All animal work was performed according to the guidelines for animal experimentation and welfare provided by Uppsala University and approved by the Uppsala County regional ethics committee (C26/15, N164-15, and 5.8.18-19429_2019).

### Data availability.

Supporting data for each figure panel are available in the supplemental Supporting Data Values file.

## Author contributions

AD, RL, and KV designed the research studies. KV, RL, PF, BDAP, MH, YS, SB, and LVH conducted experiments. KV, PF, BDAP, YS, SB, and CL acquired data. KV, RL, and AD analyzed data. FP and AKO provided resources. KV, AD, and RL wrote the manuscript. AD provided funding. RL and AD conceptualized and supervised the study.

## Supplementary Material

Supplemental data

Unedited blot and gel images

Supporting data values

## Version 1 03/05/2024

In-Press Preview

## Version 2 04/08/2024

Electronic publication
