# Supplementary material for: CD93 maintains endothelial barrier function and limits metastatic dissemination
Source: JCI Insight. 2024 Mar 5;9(7):e169830. doi: 10.1172/jci.insight.169830 (PMC11128212; doi:10.1172/jci.insight.169830)
Supplement: Supplemental data [file jciinsight-9-169830-s060.pdf]

SUPPLEMENTAL DATA FOR:

CD93 maintains endothelial barrier function and limits metastatic dissemination

Kalyani Vemuri[1], Beatriz de Alves Pereira[1], Patricia Fuenzalida[1], Yelin Subashi[1], Stefano Barbera[1], Luuk van Hooren[1], Marie Hedlund[1], Fredrik Pontén[1], Cecilia Lindskog[1], Anna-Karin Olsson[2], Roberta Lugano[1]*#, Anna Dimberg[1]*#

[1] Department of Immunology, Genetics and Pathology, Rudbeck Laboratory, Science for Life Laboratory, Uppsala University, Uppsala, Sweden

[2] Department of Medical Biochemistry and Microbiology, Uppsala University Biomedical Center, Science for Life Laboratory, Uppsala University, Uppsala , Sweden

# Equal contribution as senior authors

*Corresponding authors:

Roberta Lugano, Phone: +46709624562, Email: roberta.lugano@igp.uu.se, Address: Rudbeck Laboratory, Dag Hammarskjölds Väg 20, Uppsala University, 75185 Uppsala, Sweden.

Anna Dimberg, Phone: +46702166496, Email: anna.dimberg@igp.uu.se, Address: Rudbeck Laboratory, Dag Hammarskjölds Väg 20, Uppsala University, 75185 Uppsala, Sweden.

Conflict of interest: The authors have declared that no conflict of interest exists

SUPPLEMENTAL MATERIALS AND METHODS

Tissue microarray and image analysis

Expression of CD93, MMRN2 and fibronectin in tumor vessels was analyzed using tissue microarrays (TMAs) containing duplicate tissue cores per sample (1mm diameter) from primary lung carcinomas (adenocarcinoma, NOS (n=32); squamous cell carcinoma, NOS (n=22); large cell; NOS (n=5)), lung metastatic lesions (adenocarcinoma, NOS (n=32); squamous cell carcinoma, NOS (n=14); large cell; NOS (n=4)) and, melanoma metastatic lesions (n=20). Among the analyzed samples, 15 patient-matched sample pairs from primary and metastatic lung lesions were included (1). TMA cores were semi-quantitatively scored by two researchers in a blinded fashion on a scale of 0 to 2 (0= no staining of protein in the vessels, 1= medium intensity of the protein stained in the vessels and 2= high intensity of the protein in the vessels). Data is presented as average of 2 individual scorings. Immunohistochemical staining was performed as described (2). Briefly, slides were deparaffinized with xylene and subsequently rehydrated with absolute, 95% and 80% of ethanol respectively. Endogenous peroxidase activity was quenched with 3% H_2O_2 and the slides were boiled at 95°C in antigen retrieval buffer (pH=6) (Thermo Scientific, Waltham, MA) in a pressure cooker for 30min. Immunohistochemistry was performed with Autostainer 480 instrument (LabVision, Freemont, CA) using antibodies recognizing CD93 (HPA009300; Atlas antibodies; 1:50 dilution), MMRN2 (HPA020741, Atlas antibodies; 1:200 dilution) and fibronectin (HPA027066; Atlas antibodies; 1:25 dilution) with DAB as substrate. The stained slides were digitized with ScanScope AT2 (Leica Aperio, Vista, CA) using a 20x objective.

Cells

Parental and mCherry-tagged HCmel12 melanoma cells were generously provided by Prof. T. Tüting (Laboratory of Experimental Dermatology, University of Bonn, Bonn, Germany) and cultured in RPMI 1640 Medium (Thermofisher Scientific) supplemented with 10% fetal bovine serum (FBS). B16F10 melanoma cells (CRL-6475) and LL/2 (LLC1) Lewis lung carcinoma cells (CRL-1642) were obtained from ATCC (American type culture collection) and cultured in DMEM supplemented with GlutaMAX (Thermofischer scientific) and 10% FBS.

Human dermal blood endothelial cells (HDBECs) (Promocell) were cultured on 1% gelatin coated culture dishes in endothelial cell basal medium with full supplements (EBM-MV2; Promocell).

Primary mouse endothelial cells were isolated from the brain of 12 weeks old C57BL/6 wild-type or *CD93*$^{-/-}$ mice as previously described (3). Briefly, blood capillary fragments were seeded on rat tail collagen I coated dishes and cultured in DMEM supplemented with 20% FBS, 100µg/ml heparin and 5µg/ml endothelial growth supplements (ECGS; E2759-Sigma Aldrich). After 2days of puromycin selection (4µg/ml) cells were then used for the transwell migration assay described below.

Murine wild-type lung endothelial cells (mLECs) generated by transformation using polyoma middle T oncogene (4) were cultured in MCDB 131 (10372019; Thermofisher scientific) medium supplemented with growth factors (C-22121; Merk) and 10% FBS.

All cells were cultured at 37ºC with 5% CO_2 in a humidified cell incubator. Mycoplasma test was routinely performed in all cell cultures used in this study.

siRNA transfections

mLECs or HDBECs were incubated with scrambled control siRNA or siRNA targeting mouse CD93 (siCD93_4 and siCD93_5 for mLECs) or targeting human CD93 (siCD93_1 and siCD93_2 for HDBECs). VE-PTP was downregulated by siRNA targeting mouse VE-PTP (siVE-PTP_5 for mLEC) or human VE-PTP (siVE-PTP_5 and siVE-PTP_10 for HDBEC) (Flexitube, Qiagen) at a concentration of 2nM in a mixture of 20% Opti-MEM (Life Technologies) in MCDB cell medium supplemented with 30µl/ml Lipofectamine RNAiMAX (Life Technologies) for 4–6 hours, after which the medium was replaced with fresh medium. Experiments were performed between 48 and 72 hours after siRNA transfection.

RNA extraction and quantitative PCR

RNA isolated from blood or HCmel12 tumor tissues from wild-type and *CD93*$^{-/-}$ mice was extracted using the RNeasy Plus Mini Kit (Qiagen).

Total RNA was transcribed using iScript cDNA synthesis Kit (1708891;BIO-RAD) and mRNA expression of *m-Cherry* were quantified relative to *Hprt* by real time qPCR, in duplicate reactions per sample, with 0.25μM forward and reverse primers in SYBR green PCR master mix (Life technologies).

Primer sequences:

mCherry$_{\text{forward}}$ CCCCGTAATGCAGAAGAAGA

mCherry$_{\text{reverse}}$ TTGGTCACCTTCAGCTTGG

mouse *Hprt*$_{\text{forward}}$ CAAACTTTGCTTTCCCTGGT

mouse *Hprt*$_{\text{reverse}}$ TCGAGAGGTCCTTTTCACC)

mouse *Cldn5*$_{\text{forward}}$ TTTCTTCTATGCGCAGTTGG

mouse *Cldn5*$_{\text{reverse}}$ GCAGTTTGGTGCCTACTTCA

mouse *Cdh5*$_{\text{forward}}$ TTTCTTCTATGCGCAGTTGG

mouse *Cdh5*$_{\text{reverse}}$ GCAGTTTGGTGCCTACTTCA

mouse *Tjp1*$_{\text{forward}}$ GTTGGTACGGTGCCCTGAAAGA

mouse *Tjp1*$_{\text{reverse}}$ GCTGACAGGTAGGACAGACGAT)

mouse *Pecam1*$_{\text{forward}}$ TACTGCACGCATCGGCAAA

mouse *Pecam1*$_{\text{reverse}}$ GCATTTCGCACACCTGGAT

mouse *Mmp9*$_{\text{forward}}$ GCTGACTACGATAAGGACGGCA

mouse *Mmp9*$_{\text{reverse}}$ TAGTGGTGCAGGCAGAGTAGGA

Western blot

Cultured cells were lysed in NuPAGE LDS sample buffer under reducing conditions. The samples were separated using 4-12% Bis-Tris gel (Life technologies) and blotted on nitrocellulose membrane (Immobilon). The membranes were blocked and probed with anti-human VEGFR2 Y1175 (2478S; clone 19A10; Cell signaling Technology; 1:500 dilution), anti-human VEGFR2 (2479S; clone 55B11; Cell signaling Technology; 1:500 dilution), anti-β-actin (sc-1615; clone C4; Santa Cruz Biotechnology; 1:1000 dilution), anti-human CD93 (HPA009300; HPA; 1:500 dilution) or anti-mouse CD93 (AF1696; R&D systems; 1:500 dilution) followed by the corresponding horseradish peroxidase-labeled secondary

antibodies (Thermofisher scientific). A representative blot image of three different experiments is shown.

Co-immunoprecipitation

Co-immunoprecipitation (co-IP) was performed as previously described (5) using Pierce coimmunoprecipitation kit (Thermofishe Scientific) according to manufacturer's instructions. Briefly, total protein extract (300µg) obtained from cultured HDBECs was immunoprecipitated with 10µg mouse anti-human CD93 (D198-3; clone: mNI-11; MBL Life Science) or negative control mouse IgG (sc-2025; Santa Cruz Biotechnology). The protein concentration was measured with a commercial assay (BCA ProteinAssay Reagent kit, Pierce), as described by the providers. co-IP and flow through (FT; unbound protein fraction) samples were precipitated with ice cold acetone and re-suspended in the sample buffer for the western blot analysis.

Immunofluorescent staining of tumor sections and lungs

For immunofluorescent staining, primary tumors or lungs were embedded in OCT for cryopreservation. Samples were cut into 10µm cryosections and fixed in ice-cold acetone for 15 minutes. Sections were blocked in PBS containing 3% bovine serum albumin (BSA) for 1 hour at room temperature (RT) followed by overnight incubations with specific primary antibodies directed against CD93 (AF1696; R&D systems; 1:100 dilution), CD31 (MA3105; Clone 2H8; Life technologies; 1:100 dilution), Fibronectin (ab2413; Abcam; 1:100 dilution), Collagen IV (ab6586; Abcam; 1:200 dilution), Desmin (AF3844; R&D systems; 1:100 dilution), Ve-Cadherin (AF1002; R&D systems; 1:100 dilution), Claudin5 (352588; clone 4C3C2; Thermo Scientific; 1:200 dilution), ZO1 (61-7300; Zymed; 1:200 dilution) and MMP9 (ab38898; Abcam; 1:100 dilution), Fibrinogen (GAM/Fbg/7S; Nordic MuBio; 1:500 dilution), Glut1 (ab652; Abcam; 1:100 dilution). Sections were subsequently incubated with Alexa fluor-conjugated secondary antibodies (Invitrogen) for 1 hour at RT. Images were taken using a confocal microscope (Leica SP8) or a fluorescent microscope (Leica DMi8).

Immunofluorescent staining of mouse retina

To analyze and quantify the CD93 expression in retina vasculature, adult tamoxifen-inducible endothelial-specific knockout $CD93^{flox/flox}$ (Cre positive $CD93^{flox/flox}$), Control (Cre negative $CD93^{flox/flox}$)

as well as *CD93*$^{-/iECKO}$ (Cre positive *CD93*$^{-/flox}$) and *CD93*$^{-/+}$ (Cre negative *CD93*$^{-/flox}$) were injected 5 consecutive days with 1mg of Tamoxifen (T5648, Sigma-Aldrich) dissolved in corn oil. After 5 days of resting, retinas were dissected and stained for CD93 (AF1696; R&D systems; 1:100 dilution) primary antibody followed by incubation with Alexa Fluor–conjugated specific secondary antibody (Invitrogen). Alexa Fluor 488–conjugated isolectin B4 (I21411; Thermo Fisher Scientific) was used to visualized the vessels. A representative image of at least 3 individual retina immunofluorescent stainings per group is shown.

Immunofluorescent staining of endothelial cells

siRNA-transfected mLECs were stained for Ve-Cadherin (AF1002; R&D systems; 1:200 dilution), fibronectin (ab2413; Abcam; 1:100 dilution), Collagen-IV (ab6586; Abcam; 1:200 dilution), Claudin5 (352588; clone 4C3C2; Thermo Scientific; 1:200 dilution), Zona Occludens1 (ZO1- 61-7300; Zymed; 1:100 dilution), and MMP9 (ab38898; Abcam; 1:100 dilution). Cells were subsequently incubated with appropriate Alexa Fluor–conjugated secondary antibodies (Invitrogen). Nuclei and cytoskeleton were visualized by Hoechst; 1:5000 dilution and Alexa Fluor 647–conjugated phalloidin; 1:100 dilution, respectively (all from Life Technologies). Cells were analyzed under the confocal microscope (Leica SP8) with x63 oil immersion objective.

Tumor studies

To analyze primary tumor growth and spontaneous lung metastases formation, 9-12weeks old wild-type and *CD93*$^{-/-}$ mice were subcutaneously injected with either parental or mCherry-HCmel12 or B16F10 tumor cells ($2x10^5$cells or $2.5x10^5$ cells/100ul diluted in Dulbecco´s PBS). Mice were monitored daily and the tumors were measured by caliper twice weekly, and the tumor volume was calculated with ellipsoid formula: $V=4/3\times\pi\times(length)\times(width)\times(depth)$. After 24days from the injection, mice were sacrificed with CO_2 inhalation and tumors were excised and snap-frozen on isopentane/dry ice for cryosectioning. Lungs were perfused and fixed overnight with 4% paraformaldehyde (PFA). Subsequently, lungs were transferred into 30% sucrose solution and embedded in OCT (LAMB/OCT; Thermoscientific) for cryosection and subsequent immunofluorescent staining or alternatively, transferred into 70% ethanol and embedded in paraffin for histological analysis.

For the survival study, $2x10^5$ HCmel12 cells were inoculated subcutaneously in wild-type and *CD93*$^{-/-}$ mice. After 20 days from the inoculation of tumor cells, when the tumor reached a volume of $150mm^3$ in both groups, primary tumors were surgically resected. Mice were anestesized with isofluorane and analgesic (Rimadyl 50mg/kg) was injected subcutaneously. Primary tumors were resected and the region fo the tumor resection was properly cleaned to remove any tumor tissue remaining and the incisions sutured under anesthesia. Mice were kept under a heating lamp until awake and routinely checked post resection and sacrificed if they exhibited signs of respiratory distress, lethargy or decreased body weight.

To specifically investigate seeding of tumor cells to the lungs, mice were intravenously injected with $7.5x10^5$ HCmel12 cells or $5x10^5$ B16F10 cells in the tail vein. The body weight was measured twice weekly and mice were routinely checked for any symptoms of distress. Mice were sacrificed with CO_2 inhalation 24 days after tumor cell injection and lungs were collected and processed for histological analysis.

To evaluate the effect of inhibiting VEGF signaling, mice bearing subcutaneous HCmel12 tumors were intra-peritoneally injected with 400ug of monoclonal antibody DC101 which neutralizes mouse VEGFR2 (BE0060; BioSite) or its isotype control rIGg2a (BE0088; BioSite), both antibodies were dissolved in Dulbecco´s PBS. Mice received 4 treatments in total, starting after the observation of palpable tumors and continuing every third day. Mice were sacrificed 2 days after the last treatment and tumors and lungs were collected for analysis.

For hypoxia analysis, HCmel12 tumor bearing mice were injected with 1.5mg/100µl of hypoxyprobe-Red-549 (HP7-100 Kit) intraperitoneally 1 hour before sacrifice. Hypoxia was visualized on the cryosections with mouse Dylight-549 fluorescent antibody.

REFERENCES

1. Gremel G, Bergman J, Djureinovic D, Edqvist PH, Maindad V, Bharambe BM, Khan WA, Navani S, Elebro J, Jirstrom K, et al. A systematic analysis of commonly used antibodies in cancer diagnostics. *Histopathology.* 2014;64(2):293-305.
2. Uhlén M, Fagerberg L, Hallström BM, Lindskog C, Oksvold P, Mardinoglu A, Sivertsson Å, Kampf C, Sjöstedt E, Asplund A, et al. Proteomics. Tissue-based map of the human proteome. *Science (New York, NY).* 2015;347(6220):1260419.
3. Paolinelli R, Corada M, Ferrarini L, Devraj K, Artus C, Czupalla CJ, Rudini N, Maddaluno L, Papa E, Engelhardt B, et al. Wnt Activation of Immortalized Brain Endothelial Cells as a Tool for Generating a Standardized Model of the Blood Brain Barrier In Vitro. *PLOS ONE.* 2013;8(8):e70233.
4. Bussolino F, De Rossi M, Sica A, Colotta F, Wang JM, Bocchietto E, Padura IM, Bosia A, DeJana E, and Mantovani A. Murine endothelioma cell lines transformed by polyoma middle T oncogene as target for and producers of cytokines. *The Journal of Immunology.* 1991;147(7):2122-9.
5. Lugano R, Vemuri K, Yu D, Bergqvist M, Smits A, Essand M, Johansson S, Dejana E, and Dimberg A. CD93 promotes beta1 integrin activation and fibronectin fibrillogenesis during tumor angiogenesis. *J Clin Invest.* 2018;128(8):3280-97.

SUPPLEMENTAL FIGURES

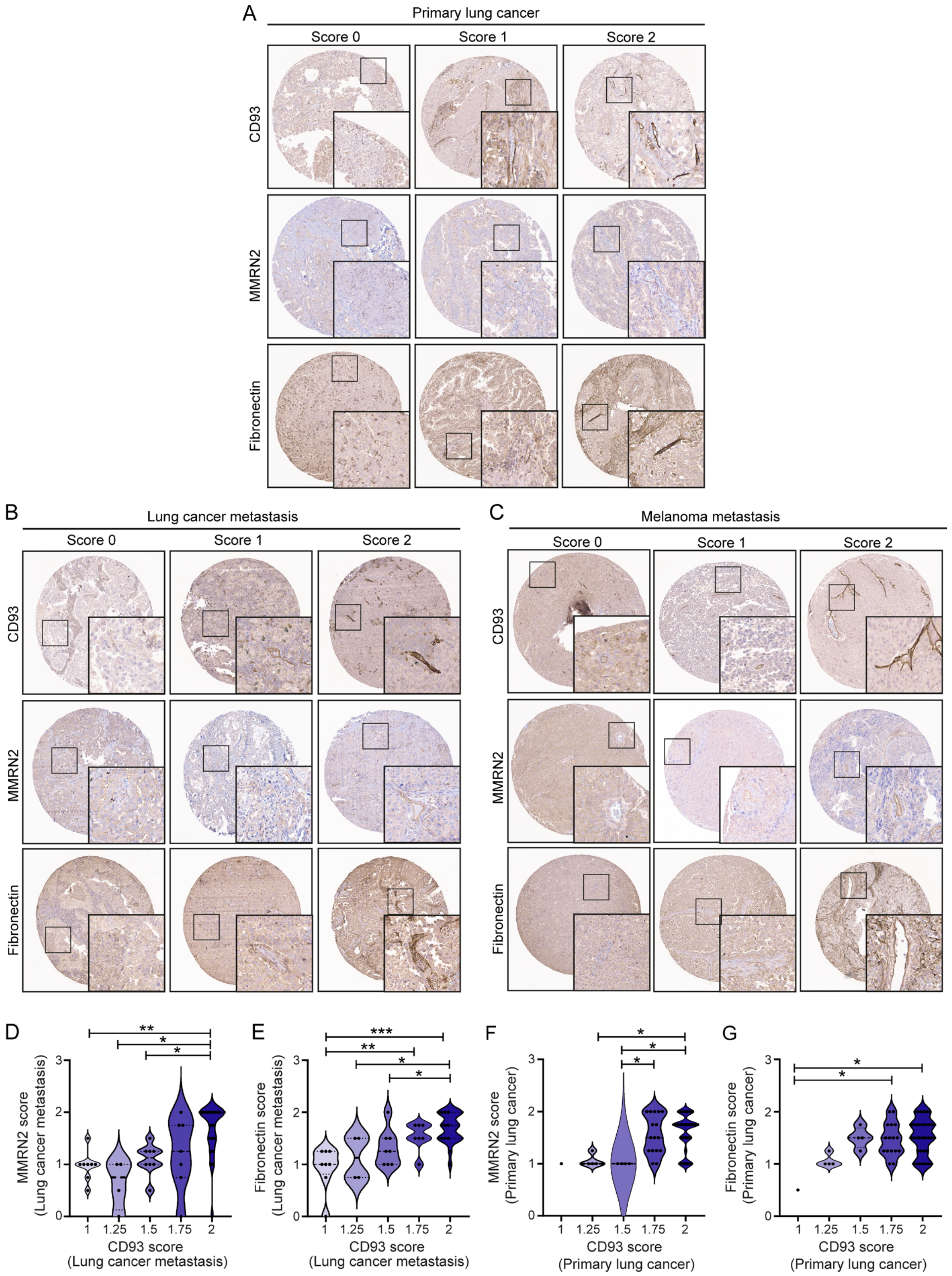

Supplemental Figure 1. Semiquantitative scoring criteria used for the analysis of human tissue microarrays and marker score comparison. (A-C) Examples of the scoring system used to quantify CD93-positive, MMRN2-positive and Fibronectin-positive vessels in the TMA (0= no staining of the marker in the vessels, 1= medium intensity of the marker in the vessels and 2= high intensity of the marker in the vessels) in the lung cancer metastasis and melanoma metastasis and in the lung carcinoma. Comparison between scoring of CD93-MMRN2 and CD93-Fibronectin in the vasculature of lung cancer metastases **(D-E)** and primary lung cancer (**F-G)** showing positive correlation of CD93 with MMRN2 and Fibronectin. * p< 0.05, **p<0.01, ***p<0.001, one-way ANOVA.

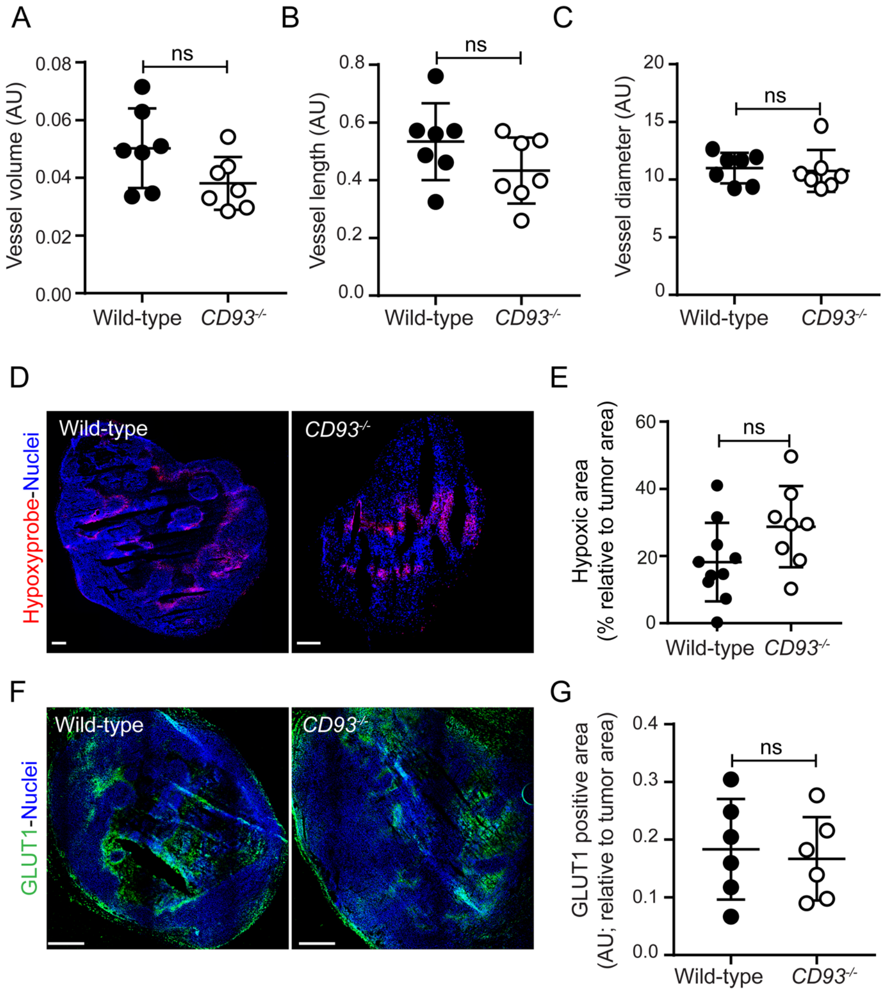

Supplemental Figure 2. CD93 deficiency does not alter tumor vessel morphology nor tumor hypoxia or apoptosis rate. Stereological quantification showing vessel density **(A)**, length (**B**) and diameter (**C**). Values are represented as arbitrary units (mean± SEM). 2-tailed *t test*. (n=7/group) **(D)** Tumor hypoxia detected by hypoxyprobe (red). Scale bars: 150µm. **(E)** Percentage of tumor hypoxic area in wild-type and *CD93*$^{-/-}$ mice (tissue tilescans of n=8-10/group). **(F)** Immunofluorescent staining for GLUT1 (green) indicating tumor hypoxia in wild-type and *CD93*$^{-/-}$ mice. Scale bars: 150µm. **(G)** Quantification of GLUT1 positive area in HCmel12 tumor tilescans of wild-type (n=6) and *CD93*$^{-/-}$ mice (n=6). Values are represented as arbitrary units (mean± SEM). ns= non-significant, 2-tailed *t test*.

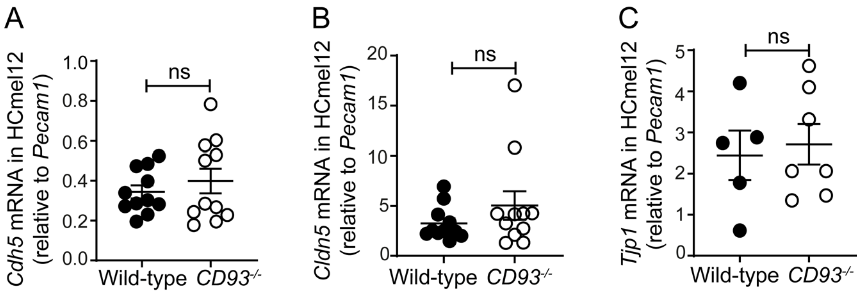

Supplemental Figure 3. CD93 deficiency does not alter mRNA levels of VE-cadherin, claudin-5 and ZO1 in HCmel12 tumors. Real time qPCR of VE-cadherin (*Cdh5*) (**A**), Claudin-5 (*Cldn5*) (**B**) and ZO1 (*Tjp1*) (**C**) showing mRNA levels in HCmel12 tumor extracts derived from wild-type and *CD93*$^{-/-}$ mice (11 mice/group for *Cdh5* and *Cldn5* and 5-7 mice/group for *Tpj1*). Values represent fold changes relative to *Pecam1* mRNA levels. ns $p>0.05$; 2-tailed *t test*. Values represent mean ± SEM.

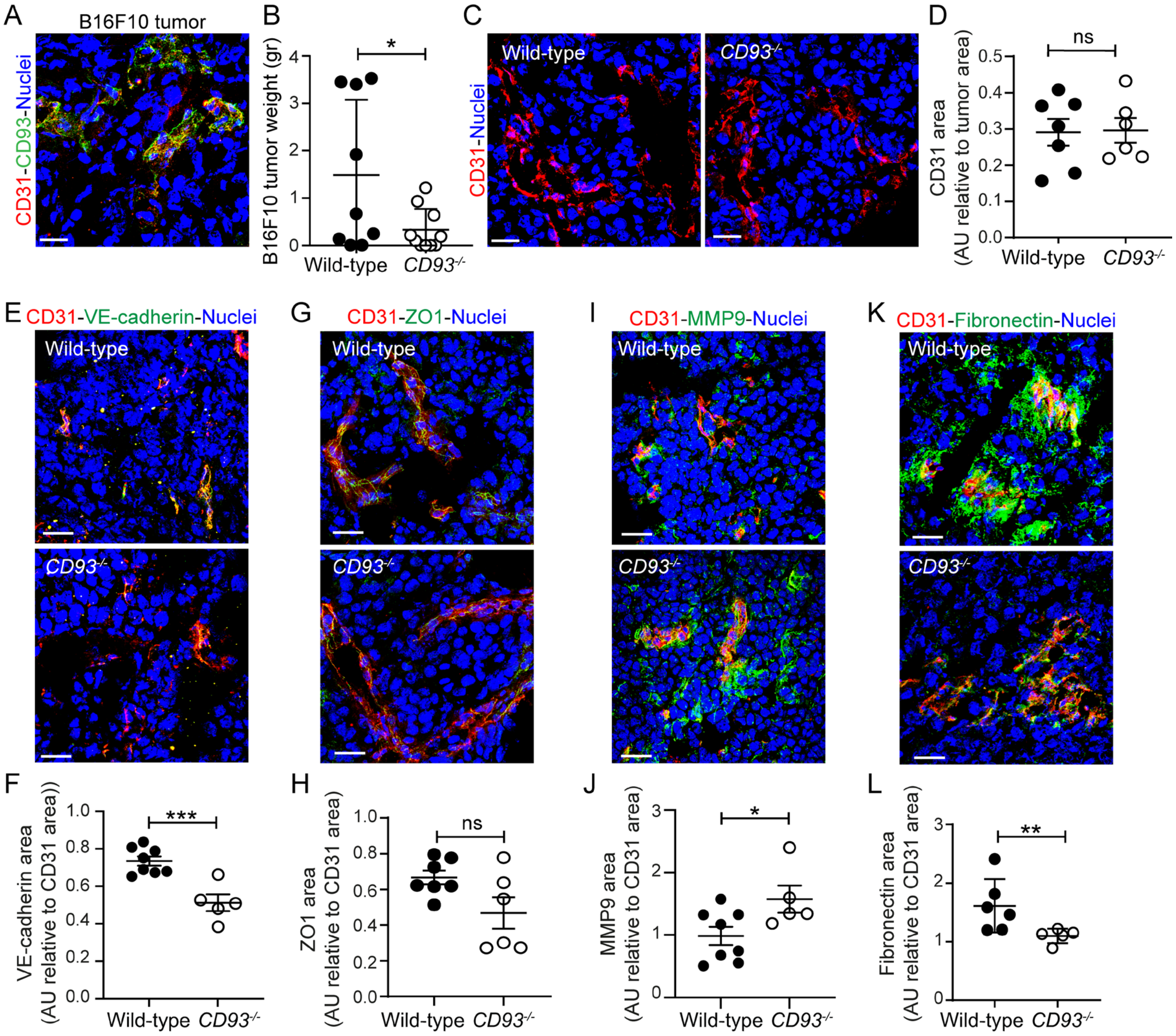

Supplemental Figure 4. CD93-deficiency impairs subcutaneous B16F10 melanoma growth and tumor vascular integrity. (A) B16F10 tumor stained for CD93 (green) and CD31 (red). Scale bars: 20µm. **(B)** Tumor weight in wild-type and *CD93*$^{-/-}$ mice (n=9-10/group). *p<0.05, 2-tailed *t test*. **(C)** Representative images of tumor vessels, CD31 (red). Scale bar: 100µm. **(D)** Quantification of CD31-positive area in wild-type and *CD93*$^{-/-}$ tumors (n=6-7/group, minimum of 3 fields of view/sample). **(E)** Representative images of VE-Cadherin (green)**, (G)** ZO1 (green), **(I)** MMP9 (green) and (**K**) fibronectin (green) in B16F10 tumors from wild-type and *CD93*$^{-/-}$ mice. (**F**, **H**, **J** and **L**) Quantification graphs of VE-cadherin (wild-type n=8, *CD93*$^{-/-}$ n=5, minimum of 5 fields of view/sample), ZO1 (wild-type n=7, *CD93*$^{-/-}$ n=6, minimum of 6 fields of view/sample), MMP9 (wild-type n=8, CD93$^{-/-}$ n=5, minimum of 8 fields of view/sample) and fibronectin (wild-type n=6, CD93$^{-/-}$ n=5, minimum of 4 fields of view/sample) levels normalized by CD31 positive area. AU=arbitrary units. *p<0.05;**p<0.01; ***p<0.001; ns=non-significant. 2-tailed *t test*. Values represent mean ± SEM.

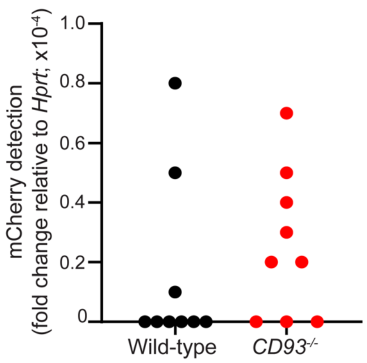

Supplemental Figure 5. Detection levels of circulating mCherry-HCmel12 cells in blood. *mCherry* expression levels was detected by real-time qPCR in the blood of wild-type and *CD93*$^{-/-}$ mice 19 days after subcutaneous injection of mCherry-Hcmel12 tumors (n=9/group). Graph represent the relative fold change of m-Cherry expression to the housekeeping gene *Hprt*.

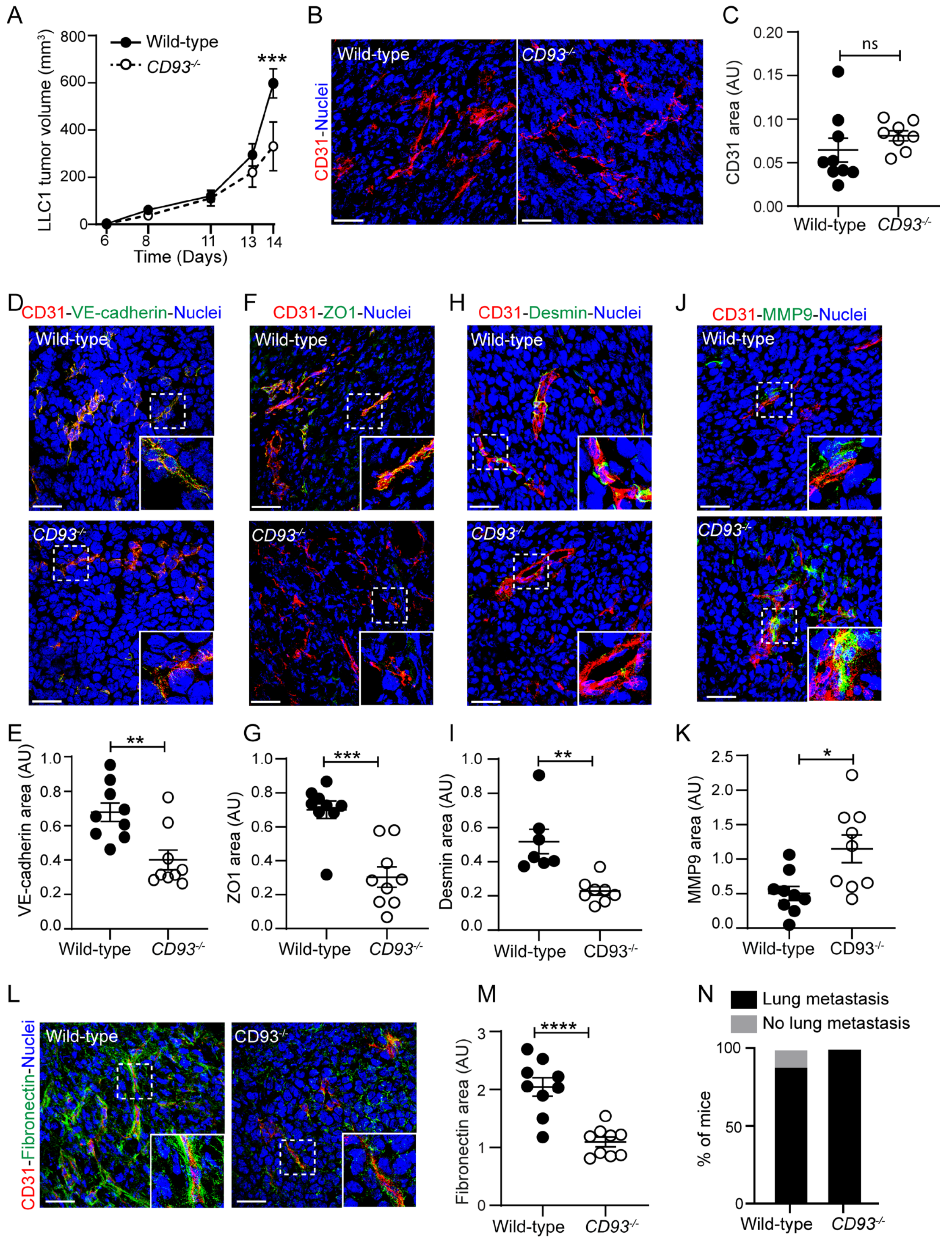

Supplemental Figure 6. CD93-deficiency impairs subcutaneous Lewis lung carcinoma growth, tumor vascular integrity and increases metastatic spread. **(A)** Tumor growth in wild-type and *CD93*$^{-/-}$ mice (n=9/group). ***p<0.001, 2-way ANOVA. **(B)** Representative images of tumor vessels, CD31 (red). Scale bar: 20µm. **(C)** Quantification of CD31-positive area in wild-type and *CD93*$^{-/-}$ tumors (n=9/group, 5 fields of view/sample). **(D)** Representative images of VE-Cadherin (green)**, (F)** ZO1 (green), **(H)** desmin, (**J**) MMP9 (green) and (**L**) fibronectin (green) in Lewis lung carcinoma (LLC1) from wild-type and *CD93*$^{-/-}$ mice. (**E**, **G**, **I**, **K**, and **M**) Quantification graphs of VE-cadherin, ZO1, desmin, MMP9 and fibronectin levels normalized by CD31 positive area (n=9/group, 5 fields of view/sample). AU=arbitrary units. *p<0.05;**p<0.01; ***p<0.001; ****p<0.0001 ns=non-significant. 2-tailed *t test*, (**N**) Lung metastasis detected in wild-type (n=9) and *CD93*$^{-/-}$ mice (n=10) subcutaneously injected with LLC1 cells 14 days after tumor inoculation.

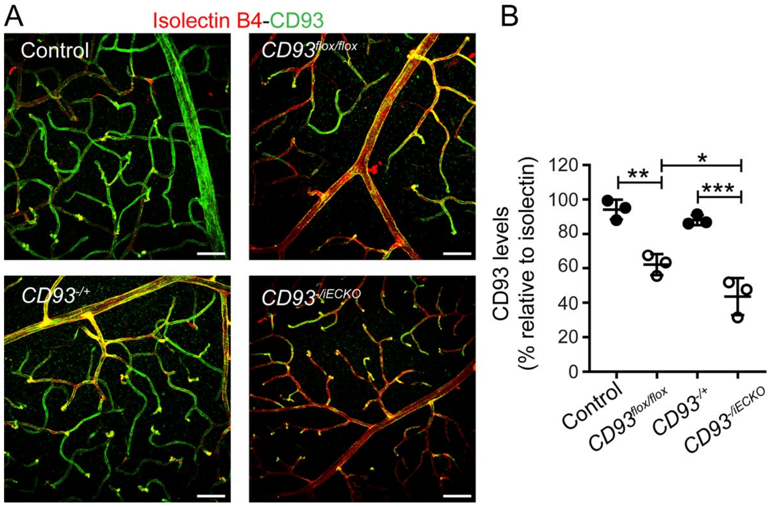

Supplemental Figure 7. CD93 levels in retina vasculature of tamoxifen-induced *CD93*$^{flox/flox}$ and *CD93*$^{-/iECKO}$ mice. (A) Immunofluorescent staining of CD93 (green) in retina isolated from tamoxifen-induced adult *CD93*$^{flox/flox}$ (*CD93*$^{flox/flox}$-*CRE*$^{+}$, n=3) and control (*CD93*$^{flox/flox}$-*CRE*$^{-}$, n=3) as well as *CD93*$^{-/iECKO}$ (*CD93*$^{-/flox}$-*CRE*$^{+}$, n=3) and *CD93*$^{-/+}$ (*CD93*$^{-/flox}$-*CRE*$^{-}$, n=3). Vessels are visualized by isolectin-B4 (red), scale bar: 50µm). (B) Quantification of CD93 levels expressed as % relative to isolectin-B4 signal. *p<0.05;**p<0.01; ***p<0.001; one-way ANOVA, Tukey´s multicomparison test. Values represent mean ± SEM.

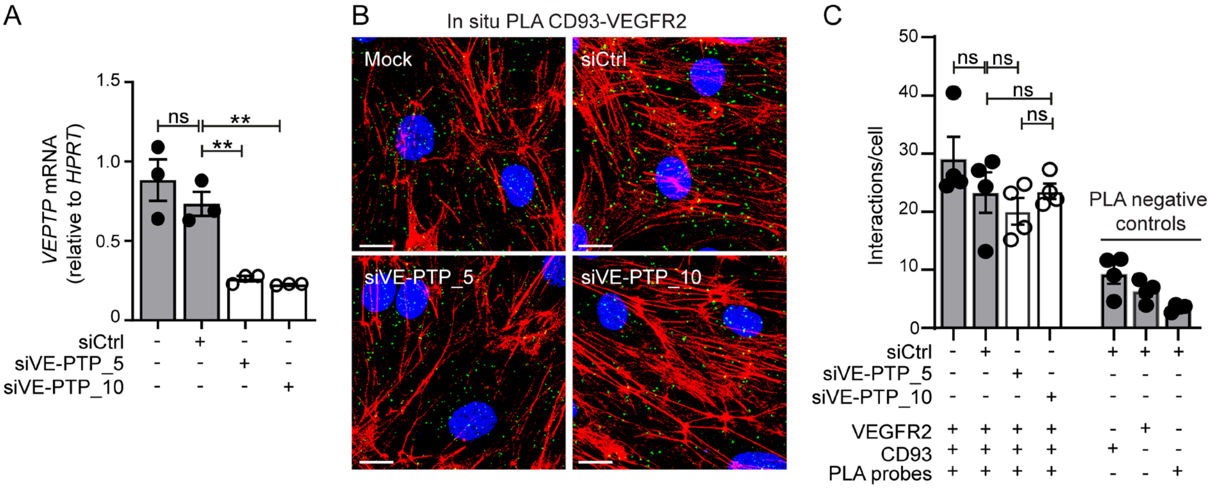

Supplemental Figure 8. VE-PTP downregulation does not affect CD93-VEGFR2 interaction. (**A**) Real time qPCR showing the efficiency of *VEPTP* knockdown in HDBECs (siVE-PTP_5 and siVE-PTP_10) at 48 hours after siRNA transfection. Untransfected cells (Mock) and scramble siRNA-transfected cells (siCtrl) were used as controls. Values represent fold changes relative to *HPRT* (3 independent experiments). (**B**) In situ PLA for CD93 and VEGFR2 in VE-PTP downregulated HDBECs. (**C**) Quantification of CD93-VEGFR2 interactions relative to cell number. Scale bar: 25µm. **<p=0.01, ns=non-significant; one-way ANOVA. Values represent mean ± SEM

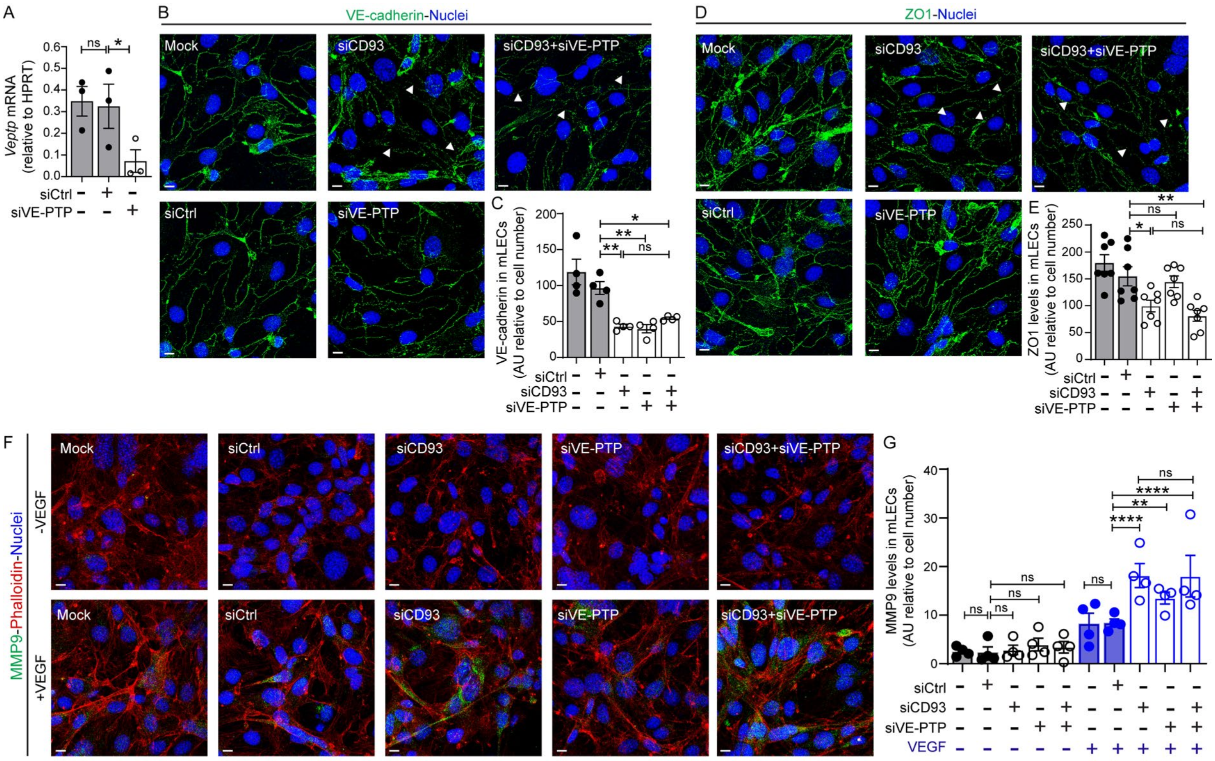

Supplemental Figure 9. VE-PTP knockdown does not further impair endothelial junctions or MMP9 levels in CD93 silenced mouse lung endothelial cells in vitro. (**A**) Real-time quantitative PCR (qPCR) showing the efficiency of *Veptp* knockdown in mouse lung endothelial cells (mLECs) (siVE-PTP) at 48 hours after siRNA transfection. Untransfected cells (Mock) and scramble siRNA-transfected cells (siCtrl) were used as controls (3 independent experiments). (**B**) Immunofluorescent staining of Ve-Cadherin (green) and (**D**) ZO1 (green) in control (Mock, siCtrl) in double silenced CD93 and VE-PTP mLECs (siCD93+siVE-PTP). Quantification of VE-cadherin and ZO1 levels (**C** and **E** respectively). (**F**) Immunofluorescent staining of MMP9 in siCD93, siVE-PTP and double silenced (siCD93+siVE-PTP) mLECs treated with or without VEGF (10ng/ml;5min). (**G**) Quantification of MMP9 levels. *p<0.05; **<p=0.01;***p<0.001; ns=non-significant; one-way ANOVA. Values represent mean ± SEM.

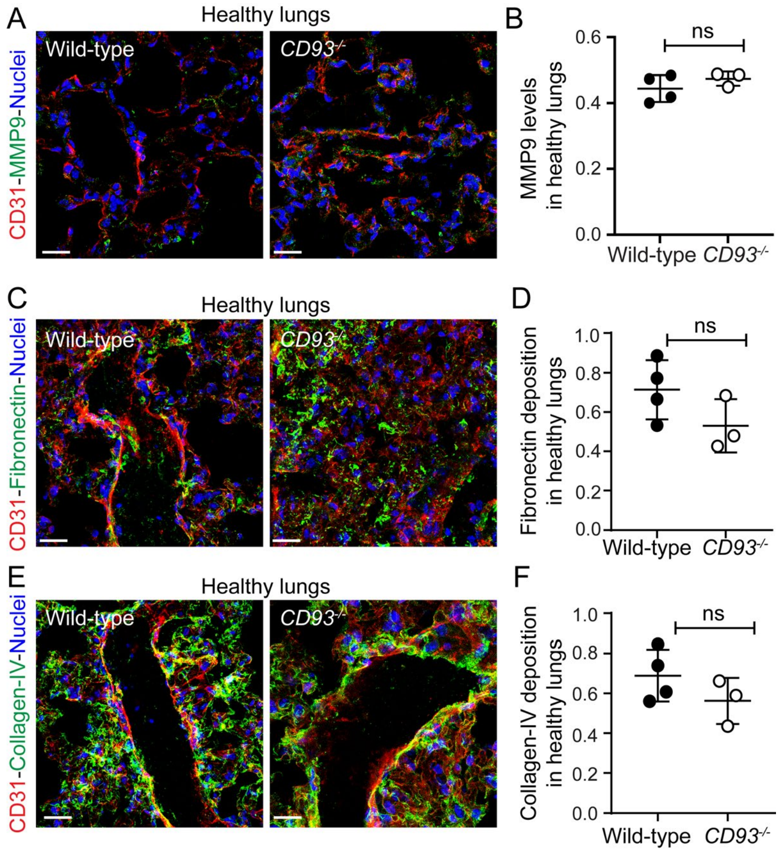

Supplemental Figure 10. CD93 deficiency does not alter MMP9 expression or extracellular matrix deposition in healthy mouse lungs. **(A-B)** Immunofluorescent staining and quantification of MMP9 (green), **(C-D)** fibronectin (green) and **(E-F)** Collagen-IV (green) of wild-type (n=4, minimum of 3 fields of view/sample) and *CD93*$^{-/-}$ (n=3, minimum of 3 fields of view/sample) mice. Vasculature is visualized by CD31 (red) and nuclei by Hoechst (blue). Scale bars: 20µm. ns= non-significant, 2-tailed *t test*. Values represent mean ± SEM.
