# Supplementary material for: CD93 maintains endothelial barrier function and limits metastatic dissemination
Source: JCI Insight. 2024 Mar 5;9(7):e169830. doi: 10.1172/jci.insight.169830 (PMC11128212; doi:10.1172/jci.insight.169830)

Full unedited blots for Figure 5A

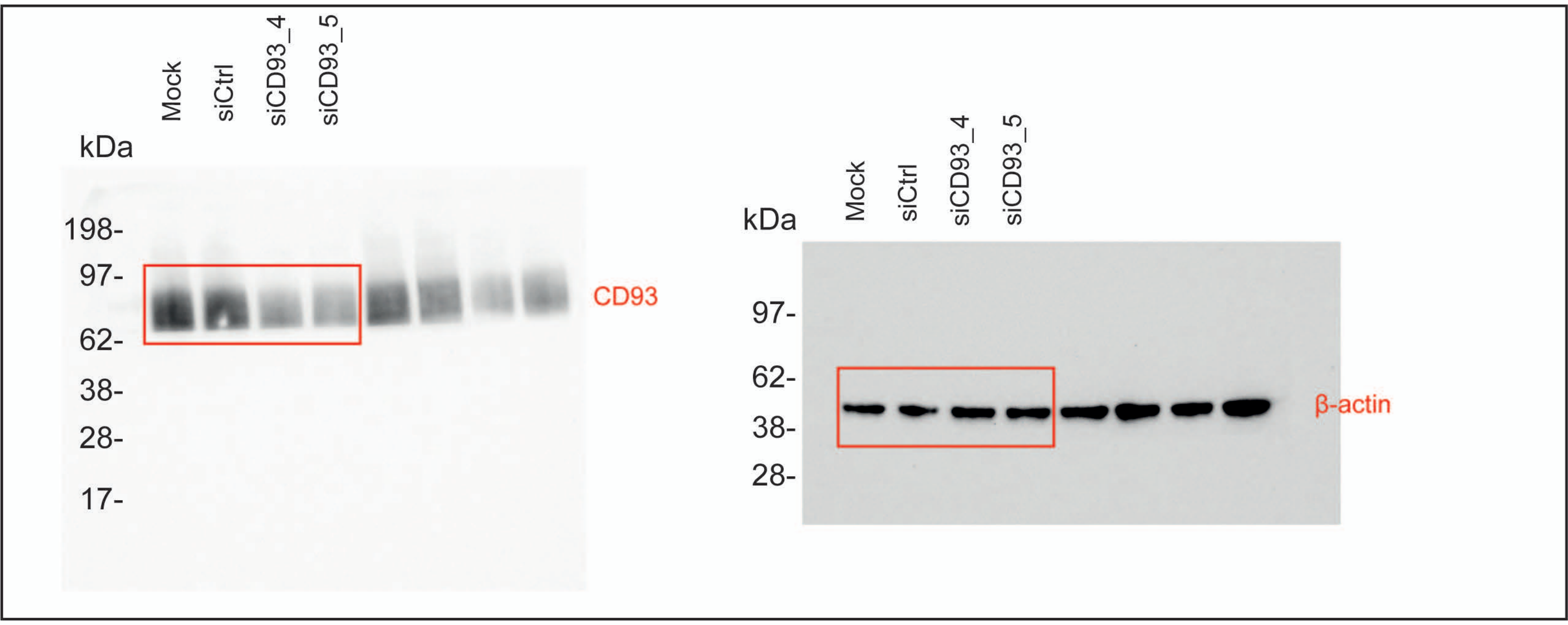

Full unedited blots for Figure 6A

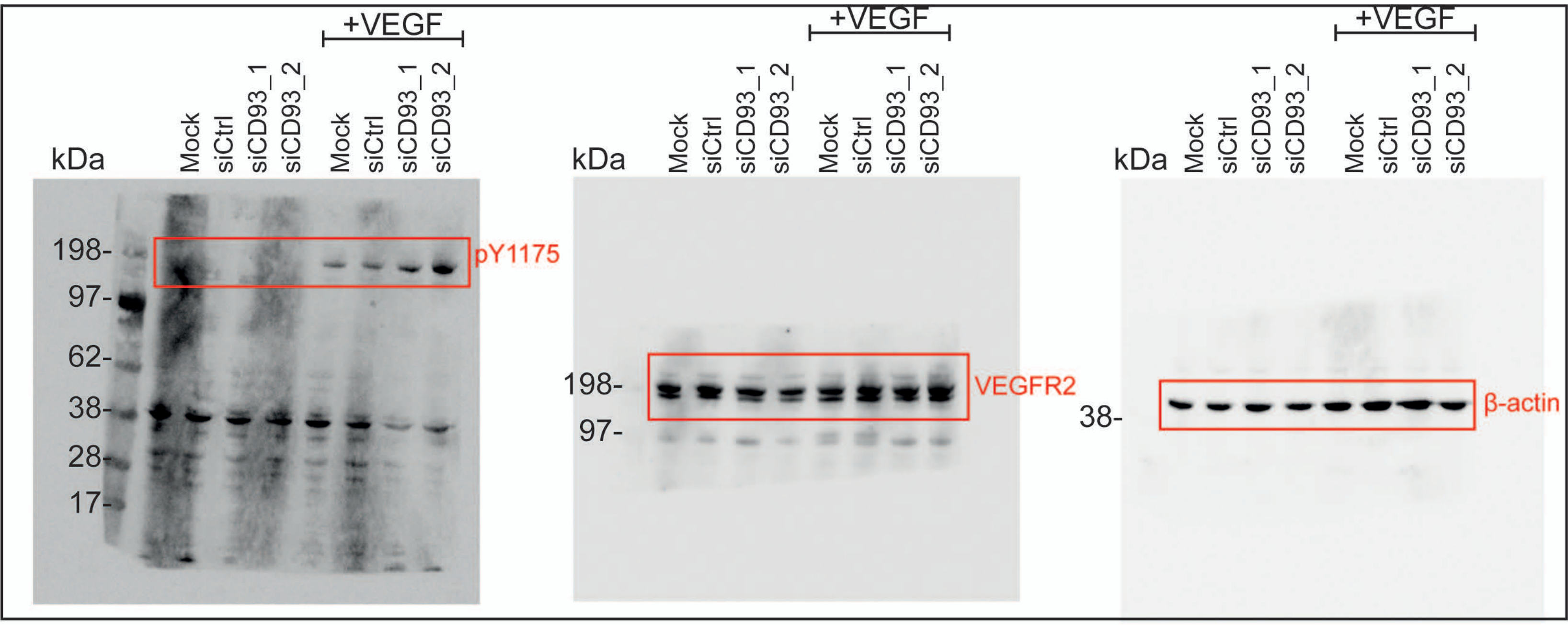

Full unedited blots for Figure 6C

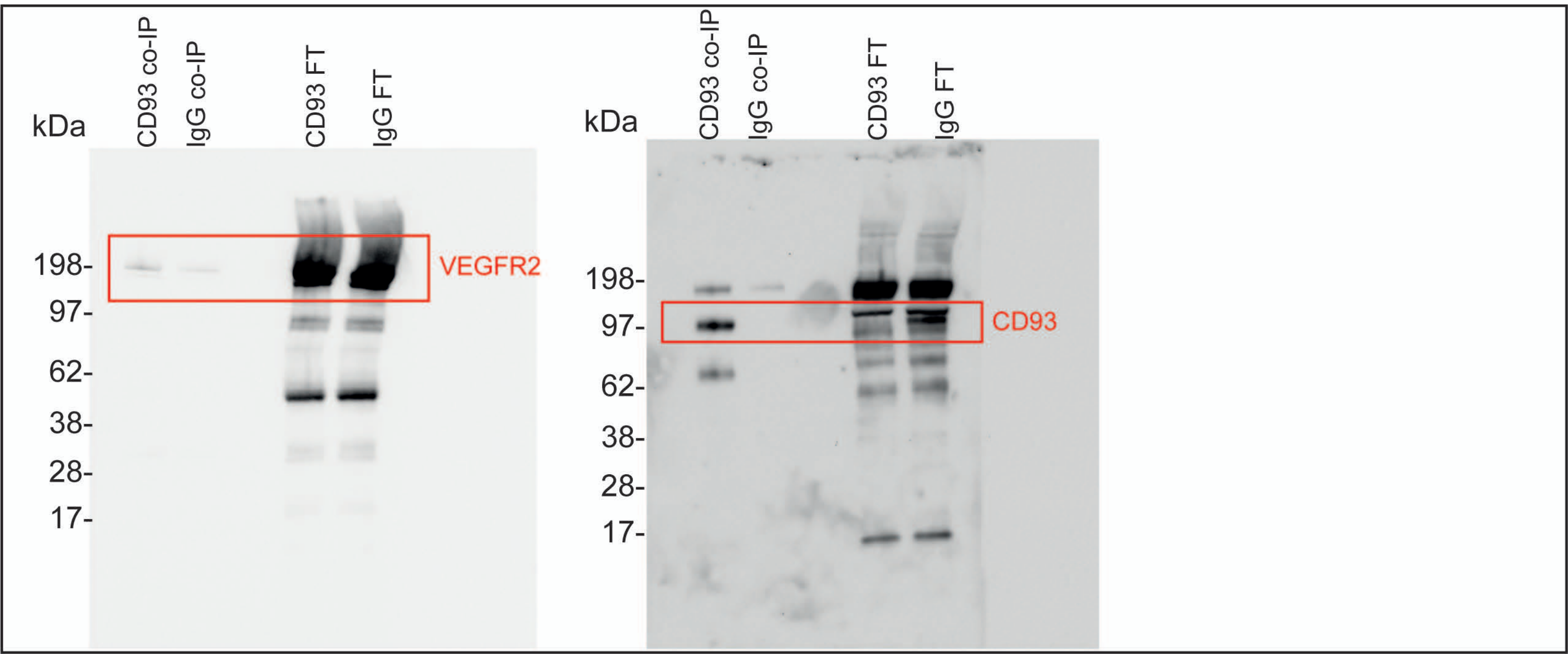

Supplement: Unedited blot and gel images [file jciinsight-9-169830-s061.pdf]
